# A knowledge graph framework for digital twins of chemical processes

**DOI:** 10.1038/s44286-026-00392-1

**Published:** 2026-05-14

**Authors:** Shuyuan Zhang, Jiyizhe Zhang, Alexei A. Lapkin

**Affiliations:** 1 Department of Chemical Engineering and Biotechnology, University of Cambridge, Cambridge, UK; 2 Cambridge Centre for Advanced Research and Education in Singapore Ltd., Singapore, Singapore; 3 Innovation Centre in Digital Molecular Technologies, Yusuf Hamied Department of Chemistry, University of Cambridge, Cambridge, UK; 4 Present Address: Department of Chemical Engineering, University of Manchester, Manchester, UK

**Keywords:** Chemical engineering, Chemical engineering

## Abstract

A digital twin, which virtually replicates a real system and fuses data, models and domain knowledge, is a key technology for accelerating chemical process development and addressing sustainability challenges. Despite its potential, one critical challenge lies in the lack of a systematic approach to integrate data, domain knowledge and predictive models to contextualize and represent chemical processes effectively. Here we propose a knowledge graph framework associated with autonomous functional agents to support the development of digital twins for chemical processes, enabling the seamless incorporation of chemical databases, artificial intelligence models and large language models. Ontologies are developed for physical models of chemical processes, allowing scalable model construction and calibration. We demonstrate the framework with practical case studies focusing on bottom-up model assembly, top-down model search and model-based reaction optimization. The framework presents an approach to manage models as a depository of chemical process knowledge, providing a foundation of digital twin technology for future chemical process development and manufacturing.

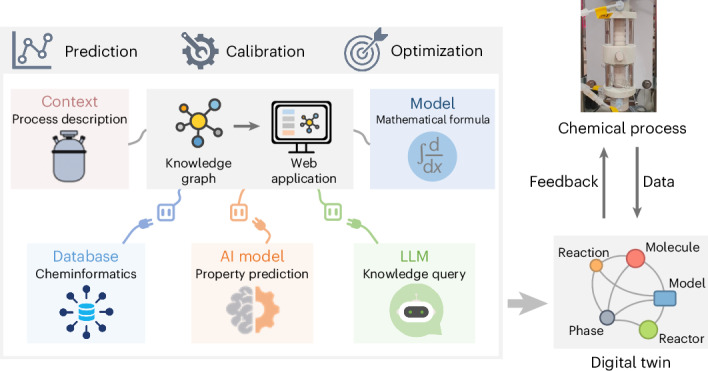

## Abstract (web summary)

This study reports a knowledge graph framework for constructing evolvable predictive physical models to support the development of digital twins for chemical processes, enabling the seamless incorporation of chemical databases, AI models and large language models. This work represents a step toward the knowledge-driven paradigm for future chemical process development and manufacturing.

## Main

The chemical industry is undergoing a rapid transition toward digitalization, as it offers solutions that deliver substantial improvements in operational efficiency, effectiveness of research and development, process safety and emissions reduction^1–3^. Digitalization at the industrial scale requires creating an architecture that links physical infrastructures—plants and laboratories—with data and knowledge^4^. The term ‘digital twin’ has emerged as a key concept in the transition to Industry 4.0^5^. Despite variations in definition across fields, it broadly refers to a continuously evolving virtual representation of a real system that fuses data, models and domain knowledge^6^. Developing digital twins today is usually not scalable, as each unit operation and process requires its unique process model and digital representation^7^. Meanwhile, there exists a significant knowledge base locked within organizations as process models that are often not consistently maintained or are unavailable due to proprietary restrictions^8^. In response to the broad and urgent need for developing digital twins, the consensus is growing to develop an approach capable of constructing and maintaining scalable digital twins, by leveraging the profound expertise and knowledge base of chemical processes^9^.

Chemical processes have long been described as complex systems^10^. To effectively capture such complexity, models are intended to serve as the backbone of digital twins to consolidate the foundation of analytical and predictive capabilities^11^. These models can be physics-based, formulated as mathematical equations^12^; data-driven, built as statistical models^13^; or a hybrid of both^14^. While statistical models are widely used and available in open-source platforms, the majority of physical models remain constrained within commercial software with restricted access^15^. These physical models are developed as solvable equations based on process descriptions and can be adapted according to certain process contexts. Once deployed, such models can be synergized with data from sensors or analytical instruments of the physical counterpart, achieving compatibility with varying process conditions through rapid model identification and calibration to act as digital twins.

Developing model-based digital twins of chemical processes requires a holistic approach that enables the rational orchestration of heterogeneous resources encompassing process descriptions and contexts, chemical databases, mathematical formulas and computational solvers, while ensuring transparent, shareable and reusable information management throughout the entire process life cycle^16^. Originating from semantic web technologies, the knowledge graph^17^ is such an approach that provides shared semantics across organizations and domains, following the FAIR principles^18^—findability, accessibility, interoperability and reusability. Although many endeavors have been made in creating knowledge graphs for chemical species^19^, reaction networks^20^ and catalysts^21^, as well as preliminary ontologies for process models^22,23^, there is still no holistic knowledge graph solution that systematically organizes a wide range of models and process knowledge for developing chemical process digital twins.

**Extended Data Fig. 1:**
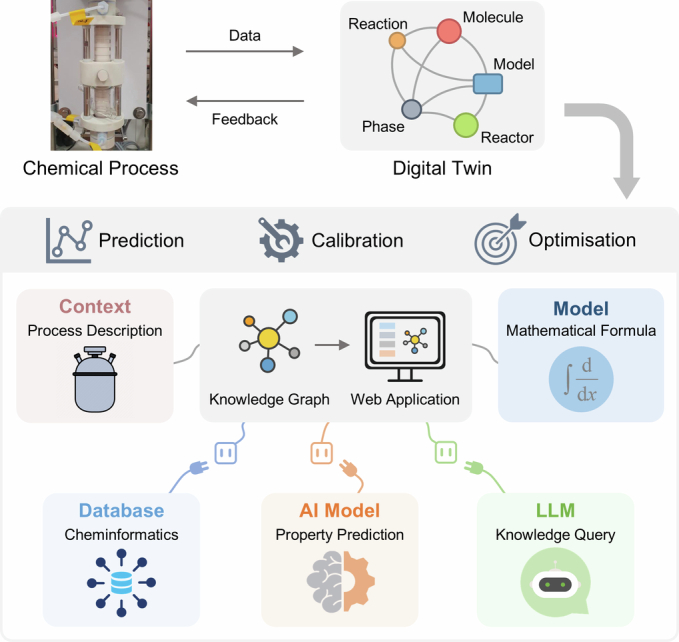
An overview of applying knowledge graph in developing physical model-based chemical process digital twins. The core of a digital twin is realised as a predictive physical model that consists of multiple modules for capturing chemical process characteristics in the aspects of molecules, reactions, phases, and reactors. Given the context of process description, the knowledge graph-based web application enables automated model identification and construction from mathematical formulas, with incorporated agents linking to databases, AI models, and LLMs.

**Extended Data Fig. 2:**
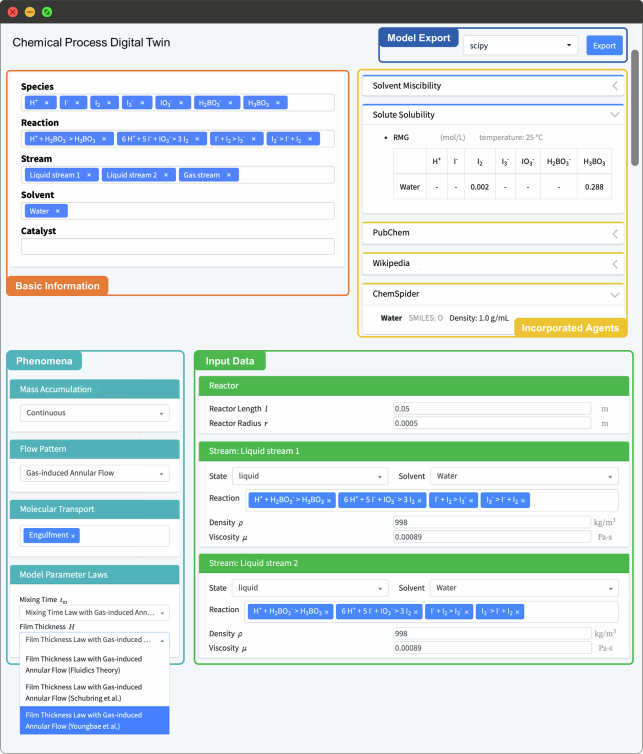
A deployed web service of our proposed knowledge graph framework. This deployed knowledge graph framework constructs predictive physical models as digital twins of chemical processes, with agents incorporated to access databases and AI models for acquiring physicochemical properties. When multiple Laws are applicable—for example, in determining film thickness in this case—a selection box is displayed to allow users to select the applied Law.

In this study, we propose a knowledge graph framework focusing on constructing evolvable predictive physical models that adapt to different scenarios to support the digital twin development of chemical processes (Extended Data Fig. 1). This framework is integrated with seamless access to up-to-date chemical databases^24^ and artificial intelligence (AI) models^25^ for physicochemical properties, as well as large language models^26^ (LLMs) for chemistry and chemical process knowledge queries through natural language. Associated autonomous agents are developed for assembling, calibrating and applying physical models of chemical processes. Both bottom-up model assembly and top-down model search approaches are realized within this knowledge graph framework for model identification and construction. As a proof-of-concept demonstration, a user interface is created to facilitate the utilization of this framework in developing chemical process models (Extended Data Fig. 2). We demonstrate the proposed knowledge graph framework by showcasing bottom-up model assembly, top-down model search and practical application in multi-objective reaction optimization for chemical processes. Ultimately, our work can be deemed as a step to support digital twin development towards the knowledge-driven paradigm for future chemical process development.

## Results

### An overview of the knowledge graph framework

The knowledge graph technology, lauded for its abilities in handling complex, heterogeneous and interrelated structures, is adopted to develop the framework for chemical process digital twins. Model knowledge and process context are ontologized separately to distinguish between foundational principles and process operational specifics. Mathematical Markup Language^27^ (MathML) is utilized to represent formulas, as it provides semantically rich, machine-interpretable mathematical expressions. Therefore, the model ontology can capture mathematical relationships between laws and variables of chemical processes, enabling hierarchical assembly of formulas into models. Databases and AI models are integrated to acquire physicochemical properties for modeling, with their application programming interfaces (APIs) represented in the model ontology to ensure unified access and interoperability. An LLM is incorporated to supplement chemistry knowledge, such as reaction information, and provide a language query entry for semantics deposited in the knowledge graph.

In detail, based on the earlier schema OntoCAPE^28^, we propose OntoModel and OntoProcess ontologies to bridge the gap between model and process knowledge in a knowledge graph framework, aiming at constructing physical models of unit operations in chemical processes.

**Fig. 1:**
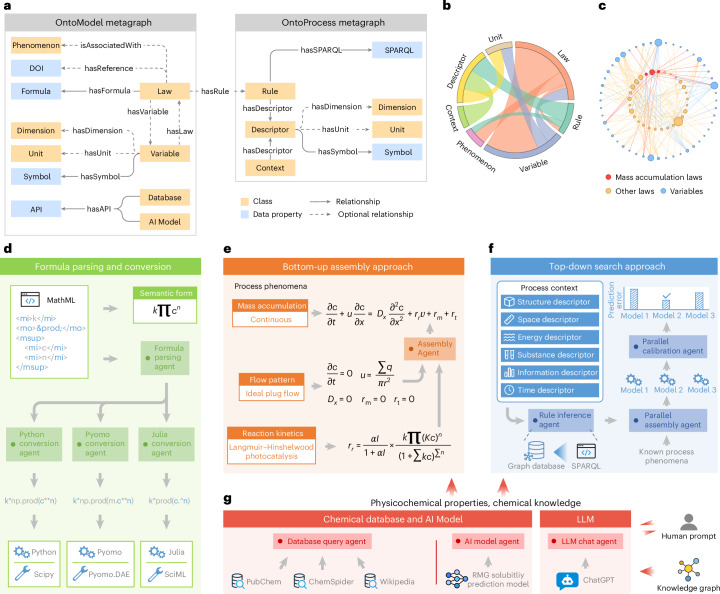
An illustration of the proposed knowledge graph framework for automated model identification and construction. **a**, Metagraphs of OntoModel and OntoProcess for representing knowledge in the ‘subject–predicate–object’ pattern. **b**, Chord diagram of OntoModel and OntoProcess pertaining to the cases involved in this work. **c**, Partial knowledge graph concerning Laws and Variables. The node radius indicates its degree in the graph. **d**, Formula parsing and conversion procedure. MathML is stored in the knowledge graph to capture mathematical formulas for constructing models compatible with various calculation packages, such as SciPy, Pyomo and Julia. **e**, Bottom-up model assembly approach. *r*_r_, *r*_m_ and *r*_t_ denote rates of reaction, molecular mixing and molecular phase transport, respectively. **f**, Top-down model search approach, with autonomous agents incorporated for rule inference, parallel construction and parallel calibration. **g**, Functional agents for accessing databases, AI models and LLMs. In this work, PubChem, ChemSpider and Wikipedia are registered to the database query agent; an AI model of the RMG software is registered to the AI model agent for predicting solubility; ChatGPT is registered to the LLM chat agent to exemplify the human–LLM interaction through prompting, with the knowledge graph preloaded as the chat context.

The metagraphs of OntoModel and OntoProcess, as illustrated in Fig. 1a, delineate the standards for defining nodes (both class and data property) and directed edges between them. In OntoModel, the Variable encompasses quantities that reflect the state or property of various entities (for example, reactor, stream, reaction, species and catalyst) attached with Dimension, Unit and Symbol to represent concepts related to matrix shape, metric measurement, and semantic expression, respectively. To depict laws for physical model construction, the Law node is introduced with three affiliated nodes: Phenomenon, which serves as the observation of chemical processes; DOI, which indicates knowledge provenance; and Formula, which expresses the mathematical relationship using MathML. APIs of Databases and AI Models also constitute a part of OntoModel to utilize chemical databases and AI models for acquiring physicochemical properties. OntoProcess is structured to present process knowledge. The process Context acts as a collective representation of the real-world chemical process, encompassing qualitative and quantitative Descriptors in all related aspects. Rule is introduced to assess the applicability of Law for chemical processes under specific operational scenarios. The inference of Rule is implemented as SPARQL codes that can be operated in a graph database engine when invoked^29^.

Following the designed metagraphs, OntoModel and OntoProcess can be populated and extended by adding model and process instances into the knowledge graph after structuration. The ontological design and an example model library are detailed in Supplementary Fig. 1 and Supplementary Tables 1–4, which includes some common formulas of generic mass balance, reaction kinetics and molecular transport. For this work, only formulas relevant to the included case studies are encoded in the knowledge graph, with the corresponding chord diagram shown in Fig. 1b. The interconnection between Law and Variable allows for programmed scanning and gathering of formulas for model construction, as depicted in the partial knowledge graph (Fig. 1c). To support the customization and extension of Law and Variable, we provide a tool to add new instances into the knowledge graph (see Supplementary Fig. 2 for details).

Figure 1d exhibits the formula parsing and conversion procedure, which is adaptable with various scientific calculation packages. MathML offers native compatibility with HTML for web display while being independent of software or hardware. Developed agents are capable of parsing and converting MathML to operable codes in SciPy, Pyomo and Julia. The detachment of formula representation and code implementation ensures the availability, functionality and consistency of the proposed knowledge graph framework in digital twin applications.

**Extended Data Fig. 3:**
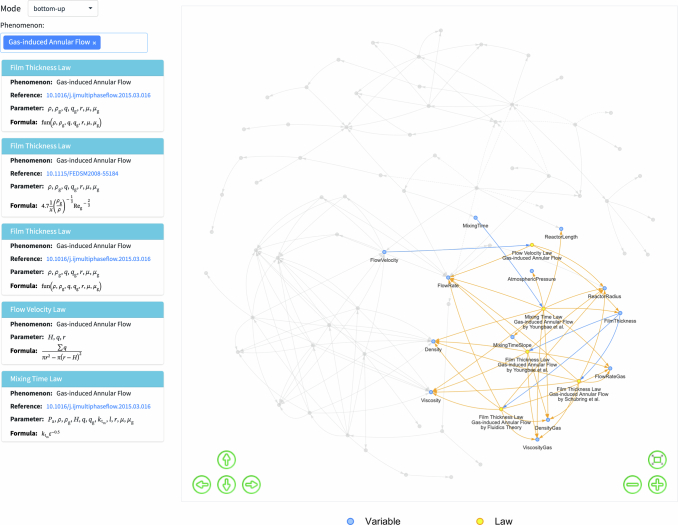
Phenomenon-induced formula retrieval on the deployed web service for the bottom-up model assembly approach. In the bottom-up approach, chemical process phenomena are looked up to retrieve formulas from the knowledge graph for model assembly. For example, formulas for calculating film thickness, flow velocity, and mixing time can be collected according to the selected Gas-induced Annular Microflow phenomenon, which are highlighted in the displayed knowledge graph.

The bottom-up assembly approach aims to construct chemical process models in the case that process phenomena are explicitly known (Fig. 1e). The mass accumulation phenomenon is first referenced to identify the overarching MathML formula that describes the net change of mass within the system. This is followed by the retrieval of other MathML expressions corresponding to flow patterns, reaction kinetics and other relevant phenomena (Extended Data Fig. 3). These collected formulas are then hierarchically assembled into a full model by the assembly agent. This modular assembly approach enables flexible adoption of custom formulas for model development in novel chemical processes. As an example, pertinent formulas of a photocatalysis process are retrieved to construct a physical model, where a photocatalyzed reaction following the Langmuir–Hinshelwood mechanism is performed in a continuous plug flow reactor with perfect radial mixing and no axial mixing^30^. Notably, in the case that multiple Laws are applicable to a Variable, the selection will be raised by the knowledge graph framework and displayed on the deployed webpage to proceed with the bottom-up assembly process, as shown in Extended Data Fig. 2.

**Extended Data Fig. 4:**
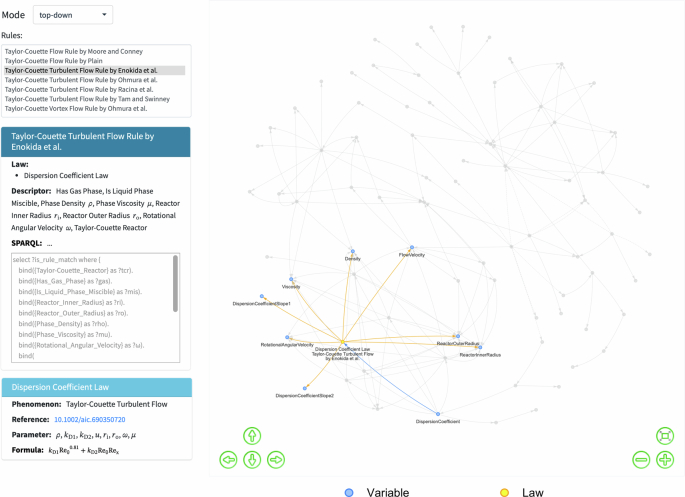
Context-induced formula filtering on the deployed web for the top-down model search approach. Rules defined in the knowledge graph are enumerated over the Process Context of the chemical process to identify applicable formulas, which are then assembled as models, calibrated with experimental data, and compared to formulate the chemical process digital twin. The Rule from Enokida et al.’s publication is illustratively selected, with the corresponding Law highlighted in the visualised knowledge graph.

The top-down search approach is designed for model identification with implicit process phenomena or indistinct formula applicability (Fig. 1f). Besides utilizing known process phenomena, this approach contextualizes the real-world chemical process, in its most feasible entirety, by gathering Descriptors in aspects of structure, space, energy, substance, information and time for the rule inference agent to enumerate OntoProcess Rules, as illustratively shown in Extended Data Fig. 4. The SPARQL codes are performed to collect Laws that match the process Context in at least one decisive Descriptor for their applicability. Subsequently, the parallel assembly agent formulates all candidate models. The parallel calibration agent then follows up to perform parameter fitting and compute prediction errors to identify the model that conforms best with experimental data.

To supplement physicochemical properties and chemistry knowledge required for model identification and construction, relevant agents are developed to harness resources such as chemical databases, AI models and LLMs (Fig. 1g). In this work, the database query agent exemplarily integrates access to PubChem^31^, ChemSpider^32^ and Wikipedia^33^ to automatically acquire the density, viscosity and miscibility of used solvents. Likewise, an AI model from the Reaction Mechanism Generator (RMG) software is adopted by the AI model agent to predict solubility^34^. Given the knowledge graph preloaded as the chat context, the cutting-edge ChatGPT^35^ is utilized by the LLM chat agent and triggered by human prompts, providing a human–LLM interface to assist with model construction. The integrated LLM is used to identify chemical reactions, as well as retrieve process phenomena encoded in the knowledge graph through language queries.

### Bottom-up model assembly for an annular microreactor

**Fig. 2:**
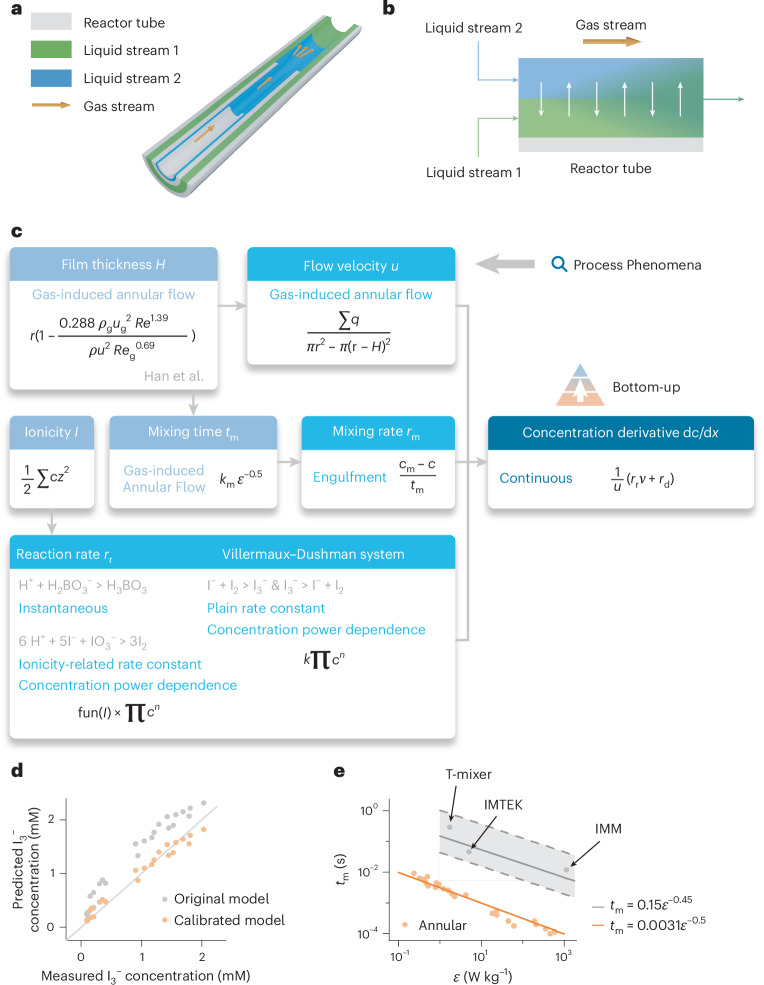
A case study of an annular microreactor for demonstrating the bottom-up model assembly approach. **a**, Structure of the annular microreactor. Three reactor tubes are coaxially aligned, with gas pumped into the innermost one to control the mixing behavior. **b**, Illustration of engulfment mixing, where streams are mixed through homogeneous molecular exchange. **c**, Bottom-up model assembly procedure. Retrieved formulas are hierarchically assembled as a physical model by an autonomous agent. **d**, Parity plots of predicted and measured I_3_^−^ concentrations for pristine and calibrated models. **e**, Mixing performance comparison of the annular microreactor with some other mixers, including T-mixer^67^, IMTEK^68^ and IMM^69^. Source data

We validate the bottom-up model assembly approach on an annular microreactor by consolidating related modeling knowledge to characterize its micromixing performance with the Villermaux–Dushman reaction^36^, as seen in Fig. 2a.

The annular reactor, consisting of three coaxially staggered quartz tubes, with gas streaming in the inner tube and liquid streaming in both middle and outer tubes, has been developed for the controllable synthesis of nanoparticles^37^ (Supplementary Fig. 3). The formation and dissipation of vortices occur at the ends of inner and middle tubes, leading to a homogenization process of concentration, known as engulfment mixing^38^. This reactor is characterized by a tunable hydrodynamic shear and therefore a controllable mixing behavior. Operation ranges and experimental data are listed in Supplementary Tables 5 and 6, respectively.

To characterize the micromixing and estimate the mixing time, the interaction by exchange with the mean model^39^ is routinely used to model engulfment mixing and is included in our knowledge graph (Fig. 2b). The empirical relationship of the film thickness has been reported by Han et al.^40^ for the gas-induced annular flow pattern. The performed Villermaux–Dushman reaction system^41^ for mixing performance characterization is as follows:

1 $${{\rm{H}}}_{2}{{\rm{BO}}}_{3}^{-}+{{\rm{H}}}^{+}\to {{\rm{H}}}_{3}{{\rm{BO}}}_{3}$$

2 $${5{\rm{I}}}^{-}+{{\rm{IO}}}_{3}^{-}+{6{\rm{H}}}^{+}\to {3{\rm{I}}}_{2}+{3{\rm{H}}}_{2}{\rm{O}}$$

3 $${{\rm{I}}}_{2}+{{\rm{I}}}^{-}\rightleftharpoons {{\rm{I}}}_{3}^{-}.$$

The kinetics can be described as follows: the acid-consuming reaction (1) is almost instantaneous; the competing reaction (2) yields I_2_ when the acid is not completely consumed by reaction (1) under poor mixing conditions; the reversible reaction (3) successively converts I_2_ to I_3_^−^, which can be measured by spectrophotometry at 353 nm.

Figure 2c illustrates the bottom-up model assembly procedure in this annular microreactor case. Known process phenomena are selected to define the model structure, followed by uploading of experimental data for model calibration. By looking up these phenomena in the knowledge graph, the assembly agent identifies a leveled hierarchy that consolidates multisource knowledge by integrating reported formulas of gas-induced annular flow pattern, engulfment mixing and Villermaux–Dushman reaction kinetics. In detail, Gas-induced Annular Flow and Engulfment phenomena lead the assembly agent to formulas of film thickness, mixing time and mixing rate in succession. The mixing time *t*_m_ is proportional to the energy dissipation rate *ε* to the power of −0.5, as suggested by Kolmogorov’s turbulence theory^42^. The film thickness is in turn used in the calculation of flow velocity. Regarding reaction kinetics, reaction (1) rate is controlled by the mixing rate according to its Instantaneous phenomenon; reaction (2) can be described with Ionicity-Related Rate Constant and Concentration Power Dependence phenomena, with corresponding formulas merged by multiplication; in contrast to reaction (2), the rate constant of reaction (3) is indicated with the Plain Rate Constant phenomenon in both forward and backward directions. Finally, the bottom-up assembly approach integrates all formulas therein into the concentration derivative formula and formulates the physical model.

Related physicochemical properties are acquired by the incorporated database query and AI model agents (Supplementary Fig. 4). The calibration agent subsequently aligns the physical model with experimental data by fitting the formula of *t*_m_. The experimental data span a wide range from 0.2 to 2.75 l min^−1^ for the air flow rate to comprehensively characterize *t*_m_. Figure 2d compares measured and predicted I_3_^−^ concentrations. The pristine model depicts the *t*_m_–*ε* relationship as *t*_m_ = 0.0075*ε*^−0.5^. However, the predicted I_3_^−^ concentration of this pristine model is constantly higher than the measurement, revealing an overestimated *t*_m_. In the annular microreactor, the *t*_m_–*ε* relationship is calibrated as *t*_m_ = 0.0031*ε*^−0.5^, delivering a good agreement between predicted and measured I_3_^−^ concentrations. Further comparison of the annular microreactor with some other mixers (T-mixer, IMTEK and IMM) of approximate geometric sizes (see details in Supplementary Table 7) confirms its superiority in achieving remarkably small mixing times with a magnitude of 0.1 ms (ref. ^43^), as shown in Fig. 2e. The calibrated physical model reveals a precisely tunable mixing time ranging from 0.1 to 10 ms in the annular microreactor.

This annular microreactor case supports the effectiveness of the bottom-up approach in physical model assembly, realizing modular, reusable and transparent knowledge management. Models can be constructed by consolidating multisource knowledge with this knowledge graph framework to represent chemical processes.

### Top-down model search for a TCR

Model screening and identification is one of the common tasks in developing new chemical processes, especially when the phenomena are not fully understood, or different assumptions can be made in modeling. Based on the proposed knowledge graph framework, a top-down model search approach is developed to address this challenge through automated model screening and evaluation.

The top-down approach is demonstrated on the Taylor–Couette reactor (TCR). A standard TCR is composed of an inner rotor and an outer cylindrical shell, forming an annular flow region for the reaction fluid in between. The inner rotor rotating speed *ω* plays a key role in determining the flow pattern and molecular transport in TCR—from mixing and dispersing induced by shear forces to plug-flow-like behavior under high flow segregation, enabling flexible mixing behavior for specific process demands^44^. However, the flow pattern and mixing behavior in TCR are not yet fully understood, and even more complex is the mixing behavior in nonstandard TCR with novel structural designs, impeding the representation of TCR.

**Fig. 3:**
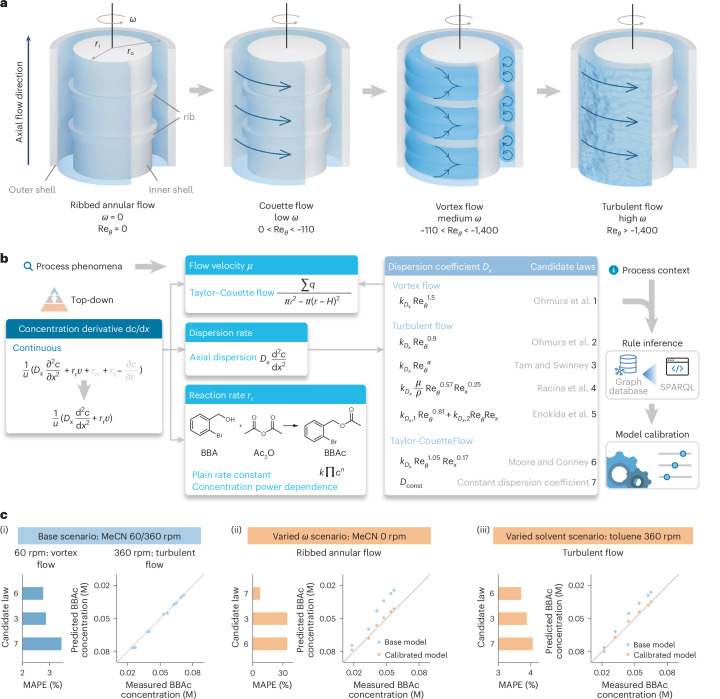
A case study of a ribbed TCR for demonstrating the top-down model search approach. **a**, Typical flow patterns in the ribbed TCR when the rotating speed *ω* increases from the stationary state, including Ribbed Annular Flow, Couette Flow, Vortex Flow and Turbulent Flow. Critical Re_*θ*_ values follow those reported in ref. ^47^, while acknowledging that they may differ for TCRs with different geometries. **b**, Top-down model search procedure for the ribbed TCR case, which performs the DMAP-catalyzed esterification reaction of BBA and Ac_2_O to form BBAc. Taylor–Couette Flow is included to denote any possible flow pattern in the TCR. Along with known process phenomena, the process Context is utilized to filter, calibrate and compare candidate dispersion coefficient Laws by the incorporated autonomous agents. **c**, Top-down model search outcomes in different operational scenarios, including (i) the base scenario with MeCN as the solvent and *ω* set to 60 or 360 revolutions per minute (rpm), (ii) the varied *ω* scenario with *ω* set to 0 rpm, and (iii) the varied solvent scenario with toluene as the solvent and *ω* kept at 360 rpm. Different flow patterns are observed in the TCR under these scenarios. The BBAc concentration is measured for model calibration. By rule inference, Laws are filtered in each scenario by running the SPARQL codes. Scenario (ii) calibrates the dispersion coefficient formula from scenario (i); in scenario (iii), the rate constant is also calibrated due to the change of solvent. Source data

TCR has been found to be critically dependent on the rotational Reynolds number Re_*θ*_ and shows different flow patterns at varying rotating speeds of the inner rotor, where axial dispersion dominates molecular transport^45^. In this case, a TCR with a ribbed inner rotor is adopted, which features the attached ribs for immobilizing vortices and influencing the overall process performance (Supplementary Fig. 5). As shown in Fig. 3a, Ribbed Annular Flow, Couette Flow, Vortex Flow and Turbulent Flow phenomena can be typically observed in this ribbed TCR as *ω* is tuned^46^, with approximate critical Re_*θ*_ values adopted from ref. ^47^. Under the laminar Couette Flow, it is found that Re_*θ*_ has little impact on the mixing efficiency. Symmetrical counter-rotating toroidal vortices would appear when *ω* is increased, indicating the onset of Vortex Flow, where both intervortex mixing (macromixing) and intravortex mixing (micromixing) are of moderate intensity and can be significantly improved by increasing Re_*θ*_. Further increases in *ω* lead to Turbulent Flow, with the mixing efficiency weakly proportional to Re_*θ*_.

An esterification reaction is performed as the example reaction, wherein 2-bromobenzyl alcohol (BBA) reacts with acetic anhydride (Ac_2_O) to produce 2-bromobenzyl acetate (BBAc). 1 mol% 4-dimethylaminopyridine (DMAP) is added as the catalyst. The rate constant is preliminarily measured as 0.051 M^−1^ s^−1^ in a flow tube with acetonitrile (MeCN) as the solvent.

Figure 3b shows the top-down model search procedure in this ribbed TCR case. Starting from the generic formula of the Continuous phenomenon, subformulas are identified iteratively by referring to known process phenomena or the process Context in the case where the phenomenon is implicit or the applicability of its corresponding Laws requires evaluation. For the dispersion coefficient *D*_*x*_ in TCR, many previous studies^48–52^ have reported correlations under certain flow patterns or as general formulas corresponding to the Taylor–Couette Flow phenomenon that denotes any possible flow pattern in TCR. A constant dispersion coefficient is also included as a degenerated Law. In the top-down search approach, the applicability of each *D*_*x*_ Law is evaluated on this ribbed TCR according to its process Context (Supplementary Table 8). Candidate models are constructed by the parallel model assembly agent, calibrated by the parallel calibration agent with experimental data, and then compared in terms of the mean absolute percentage error (MAPE) of BBAc concentration to determine the outcome model of the top-down search.

In the base scenario, MeCN is used as the solvent, and *ω* is set to 60 or 360 rpm. By rule inference, Tam and Swinney’s Law and Moore and Cooney’s Law overlap with the process Context and are parallelly calibrated, together with the constant dispersion coefficient Law^48,49^. The top-down search result turns out to be Moore and Cooney’s Law, achieving a MAPE as low as 2.8% (Fig. 3c(i)). Decreasing *ω* to 0 rpm leads to inapplicability for both reported *D*_*x*_ corrections, as the nonrotating ribbed TCR is outside the scope of those Laws (Fig. 3c(ii)). Their corresponding models degenerate into the ideal plug flow and end up with a dramatically high MAPE (34.2%). By contrast, the constant dispersion coefficient Law is revealed to deliver the lowest MAPE (7.2%) by the top-down search approach, such that the TCR model retains its predictive ability for BBAc concentration in this scenario. Switching the solvent from MeCN to toluene, while maintaining *ω* at 360 rpm, also leads to a malfunction of the model generated from the base scenario, as evidenced by the overestimated output BBAc concentration shown in Fig. 3c(iii). The top-down model search is triggered by the operational scenario change, identifying that Moore and Cooney’s Law outstands the other two, with the MAPE reduced to 3.7% after model calibration. Experimental data and calibration results under different operational scenarios are summarized in Supplementary Tables 9 and 10, respectively.

These results not only demonstrate the necessity of the top-down approach in maintaining model interoperability across a variety of operational scenarios but also highlight the capability of the proposed knowledge graph framework to comprehensively grasp the chemical process context for developing digital twins.

### Model-based multi-objective optimization of a flow chemistry system

Reaction optimization has been an everlasting pursuit in chemical process development but is often accompanied by the complexities of multidimensional parameter space, competing reaction kinetics and mass transfer effects, among others^53^. Although intuition-based methods are oftentimes taken as commonplace and data-driven methods have been attracting wide interest recently, model-based methods are favored for providing a deep understanding of the underlying chemical process mechanisms and offering reliable data extrapolation in terms of developing digital twins^54^.

**Fig. 4:**
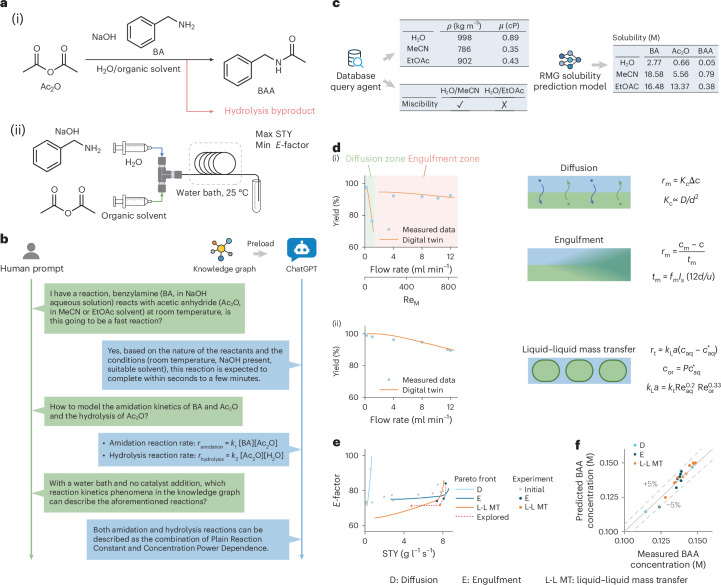
Knowledge graph-empowered model-based multi-objective optimization of an amidation reaction in flow. **a**, (i) Amidation reaction to synthesize BAA from BA and Ac_2_O, with Ac_2_O hydrolysis occurring as a side reaction. (ii) A T-mixer is used as the flow reactor to mix aqueous and organic streams while kept in a water bath at 25 °C, aiming at maximizing the STY and minimizing the *E*-factor simultaneously. **b**, Human–LLM communication for querying basic chemistry knowledge, with the knowledge graph preloaded as the chat context. **c**, Automated physicochemical property acquirement by the database query agent and solubility prediction by the incorporated AI model agent that connects to the RMG AI model. **d**, Models identified by the knowledge graph framework, with MeCN (i) or EtOAc (ii) as the organic solvent. The H_2_O/MeCN system is miscible, and its molecular transport relies on diffusion at a relatively lower flow rate and engulfment mixing at a higher flow rate; the H_2_O/EtOAc system is immiscible, for which the liquid–liquid mass transfer determines the molecular transport efficiency. **e**, Multi-objective optimization results. Constructed models are calibrated with initial experiment points designed by the maximum projection design criterion. Further experiment points guided by these models turn out near the predicted Pareto fronts. **f**, Comparison of predicted and measured BAA concentrations collected under different molecular transport mechanisms. Source data

We demonstrate the practical application of our knowledge graph framework in empowering the model-based multi-objective optimization of an amidation reaction, which is one of the essential reactions in the pharmaceutical industry for amide bond formation. In the example reaction, as illustrated in Fig. 4a, the electrophilic Ac_2_O reacts with benzylamine (BA) to synthesize *N*-benzylacetamide (BAA) in a continuous flow manner, using a reaction tube with a T-mixer. Aqueous NaOH is used as a green alternative to the commonly used organic bases but can result in the hydrolysis of Ac_2_O at the same time. MeCN and ethyl acetate (EtOAc) are optional organic solvents used to dissolve the Ac_2_O reagent, with the flow rate and electrophile concentration tuned to maximize space-time yield (STY) and simultaneously minimize the waste mass ratio (*E*-factor)^55^.

The LLM is utilized to query chemistry knowledge for developing the model of the amidation reaction in this case. Figure 4b illustrates the interaction between human and ChatGPT, where the knowledge graph is preloaded as the chat context. Basic kinetics information, as well as possible molecular transport mechanisms (Supplementary Fig. 6), can be obtained by prompting ChatGPT. The amidation reaction is found to be a fast reaction; both amidation and hydrolysis reactions are mapped to kinetics-related phenomena defined in the knowledge graph. After that, the database query and AI model agents follow up to autonomously acquire density, viscosity, miscibility and solubility information (Fig. 4c).

H_2_O/MeCN and H_2_O/EtOAc are identified as single-phase and two-phase systems, respectively. Corresponding operation ranges are given in Supplementary Table 11. Molecular transport and reaction kinetics are calibrated with initial experimental data points designed by the maximum projection design criterion^56^ (Supplementary Table 12). An obvious mixing mechanism switch from diffusion to engulfment can be observed in Fig. 4d(i). When the mean Reynolds number Re_M_ is relatively small, diffusion dominates the molecular transport and controls the reaction rate. The overall mass transfer coefficient *K*_c_ is inversely proportional to the diffusion time *d*^2^/*D*, where *d* and *D* denote hydraulic diameter and diffusion coefficient, respectively^57^. According to the Rule reported by Schikarski et al.^58^, the transition occurs when Re_M_ is approximately 160. The corresponding Law of *t*_m_ for engulfment is disclosed as *f*_m_*I*_s_(12*d*/*u*), where *u* represents the flow velocity, *I*_s_ the segregation intensity and *f*_m_ a tunable parameter. Calibration results of identified models are presented in Supplementary Table 13.

In the H_2_O/EtOAc system, molecular transport mainly relies on liquid–liquid mass transfer. The Law published by Li et al.^59^ is retrieved from the knowledge graph, which calculates the volumetric mass transfer coefficient *k*_L_*a* as *k*_t_*Re*_aq_^0.2^*Re*_or_^0.33^, where *Re*_aq_ is the Reynolds number of the aqueous stream, *Re*_or_ the Reynolds number of the organic stream and *k*_t_ a tunable parameter (Fig. 4d(ii)). The BAA yield decreases only moderately with increasing flow rate due to the reasonably efficient mass transfer in the two-phase system.

The calibrated models are then applied to calculate Pareto fronts under different molecular transport mechanisms to guide the multi-objective optimization campaign, as shown in Fig. 4e. The plot reveals that engulfment and liquid–liquid mass transfer reach the most bottom-right region in the plot, indicating the best trade-off between STY and *E*-factor in prediction. Following these results, subsequent experiments are sampled along the predicted overall Pareto front, which consists of two arcs respectively dominated by engulfment and liquid–liquid mass transfer phenomena, to validate the effectiveness of constructed physical models in reaction optimization (Supplementary Fig. 7). The resultant experiment points are near the model-predicted Pareto fronts for both engulfment and liquid–liquid mass transfer, showing commendable agreement between model-based optimization results and experiments. The final experimental Pareto fronts are given in Supplementary Table 14. In addition, the parity plot of all data points delivers a MAPE below 5% (Fig. 4f). In addition, this physical model-based optimization method is benchmarked against Bayesian optimization^60^ on a yield maximization task for a virtual nucleophilic aromatic substitution reaction^61^ (Supplementary Fig. 8). It substantially outperforms the data-driven Bayesian optimization baseline, highlighting the advantage of incorporating chemistry and mass transfer phenomena into reaction optimization. These results demonstrate that our knowledge graph framework can effectively leverage the predictive ability of physical models and facilitate model-based multi-objective reaction optimization.

## Discussion

In this work, we develop a holistic knowledge graph framework for developing predictive models that support model-based digital twin development of chemical processes. This knowledge graph framework enables modular, reusable and transparent knowledge management, enabling the incorporation of functional autonomous agents for model assembly and calibration, rule inference, database access, AI integration and LLM utilization. Both bottom-up and top-down approaches are realized to construct models based on the knowledge graph framework.

While there is growing interest in knowledge-driven scientific methods, a key bottleneck remains the systematic and extensive representation, integration and standardization of modeling knowledge for chemical processes. Accompanying this challenge are issues related to data availability, including heterogeneous formats, limited machine interoperability and the lack of semantically rich metadata. This work presents a knowledge graph infrastructure to advance the conceptualization for high-level chemical process description by developing process phenomenon and context classes as the foundational knowledge base for chemical engineering, designed to interface seamlessly with experimental data and modeling information. Former phenomenological methods for process design and intensification are referred to in defining the phenomenon class of the knowledge graph, which can enable investigation of the whole design space with superstructure optimization^62,63^. Meanwhile, our work also covers the context-based description, of which the concept is rising recently for matching models with process data, integrating life-cycle information and addressing scale-up problems in chemical process development^64,65^. This knowledge graph framework enlightens an approachable way to systematically integrate model and process knowledge, facilitating the development of digital twins of chemical processes, with accessible links to up-to-date databases and AI tools. At present, the LLM chat agent is used to supplement chemistry knowledge and reformulate existing entities within the knowledge graph without domain-specific retraining. The capabilities of the LLM agent can be further enhanced by incorporating reaction ontologies, fine-tuning in the chemical engineering domain and enabling phenomenon inference that accounts for reactor geometry and operating conditions via prompting. Advancing AI tools capable of populating and expanding chemical process knowledge also offers a promising direction for future development of the framework. This framework is promising to effectively deposit, manage, populate and reuse both existing and cutting-edge chemical process knowledge.

The use of AI in combination with leveraged data and knowledge offers the most efficient route toward scalable digital twins and the emergence of fully digital chemical manufacturing. Besides the developed digitalization tool, we perceive that the proposed framework can be further developed as a backend for future remote education, online training and multi-organizational collaboration in complex technical subjects of chemical processes, overcoming spatial limitations and lowering the access barrier to chemical process knowledge while maintaining a systematic knowledge management structure. A further direction for developing this knowledge graph framework is to discover new first-principles knowledge by inferring subgraph motifs that relate phenomena, laws and variables, which can greatly benefit from technologies such as symbolic regression. We expect that the proposed knowledge graph framework, along with associated autonomous agents therein, will become a strong support for chemical engineering in the future.

## Methods

### Knowledge graph framework development

The knowledge graph practice in this work follows the Web Ontology Language (OWL), which is a semantic web language designed to represent rich and complex knowledge about things, groups of things, and relations between things. The API of OWL (https://github.com/owlcs/owlapi) is applied for creating, manipulating and serializing OWL ontologies. The rich semantics embedded in the knowledge graph allow users to focus on the intelligent work of defining the model structure, through either the bottom-up or the top-down method, while delegating parameter identification, formula assembly and code generation to developed agents. The ontological knowledge graph development is presented below, which can be referred to for further development of the knowledge representation for chemical processes.

#### Ontology development

The development of ontologies for a specific domain always follows the pathway from conceptualizing entities and relationships as target deliverables to implementing detailed codes. We reuse an existing practice, OntoCAPE, to further develop and extend ontologies related to chemical engineering. We also draw inspiration from available software tools, conceptual publications and existing packages. Variable and Law are two core entities in our ontology framework, which are utilized to describe chemical process states and the underlying laws. Their related concepts are also abstracted as entities in the knowledge graph with connections to them. In the bottom-up approach, Phenomenon is provided for retrieving Laws following relationships in the knowledge graph, whereas the top-down search approach also adopts Context to validate Laws coupled with Rules and determine the applicability. It is noted that the DOI, API and SPARQL are defined as xsd:string, while Symbol and Formula are defined as rdf:XMLLiteral to represent mathematical notations. Extracted knowledge can be added to the knowledge graph in a plug-and-use manner by constructing relevant entities and relationships. The consistency of ontologies is verified using the HermiT reasoner^66^ in the Protégé software.

#### Autonomous agent development

To effectively utilize the aforementioned knowledge graph, agents are developed to conduct certain tasks. Agents are programmed to autonomously explore the knowledge graph and process extracted information as output, provided that access to entities and relationships is available. Aiming at digital twin application, we build agents for (parallel) model assembly, (parallel) model calibration, rule inference, database query and AI model invocation. To incorporate these agents into the web deployment, we adapt these agents into API services in a web server. This allows for the Hypertext Transfer Protocol (HTTP) request to trigger deployed agents and obtain desired responses from the backend.

In detail, the (parallel) model assembly agent is for the retrieval and integration of Laws as a digital twin based on the given Phenomenon (Supplementary Fig. 9). The (parallel) model calibration agent is designed to operate the constructed digital twin and optimize parameters assigned in the HTTP request with given upper and lower limits (Supplementary Fig. 10). The parameter fitting is realized by minimizing the concentration root mean square error through the differential evolution optimization algorithm in SciPy. The rule inference agent is to run the SPARQL code in a graph database to determine whether the corresponding Law is suitable for the specific scenario (Supplementary Fig. 11). The database query agent and the AI model agent are set up to autonomously acquire physicochemical properties related to the given chemical process in the HTTP request.

#### Web deployment

A web deployment is developed to facilitate human–cyber interaction by providing a functional user interface, as illustrated in Supplementary Fig. 12. The ontological knowledge graph can be, therefore, promisingly empowered by versatile resources on the Internet. The web deployment follows a routine practice for cloud applications and is containerized using Docker (https://www.docker.com). The separation of concerns principle is followed to break down the web service into two sections: knowledge graph and web service. For knowledge graph deployment, a Docker image is built based on the free version of GraphDB (https://graphdb.ontotext.com) with entities and relationships preloaded. Meanwhile, the web service is realized with the Flask (https://flask.palletsprojects.com) framework and containerized into another Docker image. Overall, this deployment allows the detachment of the knowledge graph and the web service, providing an accessible portal for applications. Our knowledge graph framework is illustratively deployed at https://kg4dt.kg.cdi-sg.com/app/index.

The webpage allows users to define the basics of species, reactions, streams, solvents and catalysts for the chemical process; then, autonomous agents at the backend will automatically acquire and contextualize chemical information and physicochemical properties from databases, AI predictive models and LLMs. Available information and data are then supplemented on the webpage for agents to assemble, calibrate and export physical models compatible with multiple solvers. This webpage-based knowledge graph deployment underpins the potential of a digital platform to integrate multisource resources through APIs for constructing digital twins.

### Experimental methods

Three cases are adopted in this work to demonstrate the effectiveness of the proposed knowledge graph framework in bottom-up model assembly, top-down model search and practical application in multi-objective reaction optimization, respectively. The respective reaction systems are an annular microreactor with the Villermaux–Dushman reaction, a ribbed TCR with an esterification reaction, and a T-mixer with an amidation reaction. The experiment description is provided below. Experimental data and details can be found in the Supplementary Information.

#### Annular microreactor case

The commonly used Villermaux–Dushman reaction system is applied to measure the mixing time of the annular microreactor. Three quartz tubes are installed in a tube-in-tube configuration. With gas streaming in the inner tube, a thin film would form at the end of the inner tube; mixing and reaction would occur after the end of the middle tube. A KDS Legato Dual Syringe Pump was used to deliver liquids in plastic 10-ml or 50-ml Terumo syringes. A Sierra SmartTrak C50L Mass Flow controller was used to deliver air with a 200-µm filter (Swagelok) installed, with a maximal flux of 20 l min^−1^ and an accuracy of 2%. From inner to outer, sizes of quartz tubes purchased from VitroCom are 0.3 mm inner diameter (ID) × 0.4 mm outer diameter (OD) × 100 mm length (L), 0.5 mm ID × 0.7 mm OD × 100 mm L, and 1 mm ID × 1.2 mm OD × 300 mm L. The reaction zone length was set to 50 mm. See more details about the assembly of this custom-built annular reactor in the Supplementary Information. The reaction competition in the Villermaux–Dushman reaction system can indicate the mixing efficiency. Potassium iodide, potassium iodate, boric acid, sodium hydroxide and sulfuric acid were purchased from HCS Scientific. A solution of 0.25 M H_3_BO_3_, 0.125 M NaOH, 0.005 M KI and 0.001 M KIO_3_ and an acid solution of 0.05626 M H_2_SO_4_ were prepared at room temperature. Flow rates of the two solutions were equal and ranged from 0.4 to 20 ml min^−1^. The air flow rate ranged from 0.2 to 2.75 l min^−1^. The product was diluted, if necessary, and analyzed by an ultraviolet (UV)–visible light absorption test using an Agilent Cary 60 at 353 nm to measure the concentration of triiodide (I_3_^−^).

#### Ribbed TCR case

An esterification reaction of BBA and Ac_2_O is conducted in a ribbed TCR (Autichem) to synthesize BBAc, of which the ^1^H NMR spectra is shown in Supplementary Fig. 13. Experiments were conducted at room temperature (25 ± 1 °C). A solution of BBA (0.2 M, 1 equiv.), triethylamine (0.2 M, 1 equiv.) and DMAP (0.002 M, 0.01 equiv.) was injected into the ribbed TCR together with another solution of Ac_2_O (0.2 M, 1 equiv.) by two pumps (Vapourtec R series) with polytetrafluoroethylene tubes. The solvent (MeCN or toluene) was set to be the same in the two streams. After the ribbed TCR achieved a steady state, the outlet liquid was collected and quenched using 1 M HCl solution. The reaction sample was then diluted and sent to high-performance liquid chromatography (HPLC)-UV for analysis, with HPLC calibration curves shown in Supplementary Figs. 14 and 15.

#### Amidation reaction in a flow system

An amidation reaction was conducted in a continuous-flow system. A polytetrafluoroethylene T-mixer (0.5 mm ID) was connected to a reaction tubing (0.5 ml) and was immersed in a water bath at 25 °C. Ac_2_O wa dissolved in MeCN or EtOAc, while BA and NaOH were dissolved in water. The two solutions were injected separately into a T-mixer using two syringe pumps (TriContinent). The resultant mixture was collected into 1 M HCl aqueous solution in a sample vial for quenching the reaction. After dilution of the reaction mixture, the concentration of the product BAA was determined by HPLC-UV (Shimadzu LC-20AD Liquid Chromatograph), using the HPLC calibration curve plotted in Supplementary Fig. 16. For optimization, the initial concentration of BA was set to 0.3 M. NaOH and Ac_2_O concentrations were set to the same, with the molecular equivalent ranging from 1.0 to 1.5. The flow rate of each pump could vary from 0.10 to 6.00 ml min^−1^, corresponding to a residence time of 2.5–150 s. The initial points included a series of experiments with a consistent concentration of 0.39 M for NaOH and Ac_2_O. A few points with varied concentrations of NaOH and Ac_2_O were also added to the initial points.

## Supplementary information

Supplementary Information Supplementary Triples 1 and 2, Figs. 1–16 and Tables 1–14.

## Source data

Source Data Fig. 2 Measured and predicted mixing performance data of the annular micromixer, as well as those of other mixers including T-mixer, IMTEK and IMM.

Source Data Fig. 3 BBAc prediction results of the TCR case under different scenarios and MAPE of candidate laws.

Source Data Fig. 4 Measured data and reaction optimization results of the model-based multi-objective optimization case.

## Data Availability

Experimental data are available in the Supplementary Information and via GitHub at https://github.com/sustainable-processes/KG4DT. Source data are provided with this paper.

## Code availability

All codes in this study are available via GitHub at https://github.com/sustainable-processes/KG4DT. The docker file for deploying the knowledge graph in GraphDB is developed from https://github.com/khaller93/graphdb-free.

## References

[1] Torrente-Murciano, L. et al. The forefront of chemical engineering research. *Nat. Chem. Eng.* **1**, 18–27 (2024).

[2] Meng, F. et al. Planet-compatible pathways for transitioning the chemical industry. *Proc. Natl Acad. Sci. USA* **120**, e2218294120 (2023). 36787351 10.1073/pnas.2218294120 PMC9974437

[3] Zuin Zeidler, V. Digitalization paving the ways for sustainable chemistry: switching on more green lights. *Science* **384**, eadq3537 (2024). 38870287 10.1126/science.adq3537

[4] Fantke, P. et al. Transition to sustainable chemistry through digitalization. *Chem* **7**, 2866–2882 (2021).

[5] Tao, F., Zhang, H. & Zhang, C. Advancements and challenges of digital twins in industry. *Nat. Comput. Sci.* **4**, 169–177 (2024). 38532139 10.1038/s43588-024-00603-w

[6] Tao, F. & Qi, Q. Make more digital twins. *Nature* **573**, 490–491 (2019). 31554984 10.1038/d41586-019-02849-1

[7] Hao, Z., Barecka, M. H. & Lapkin, A. A. Accelerating net zero from the perspective of optimizing a carbon capture and utilization system. *Energy Environ. Sci.* **15**, 2139–2153 (2022).

[8] Davis, J., Edgar, T., Porter, J., Bernaden, J. & Sarli, M. Smart manufacturing, manufacturing intelligence and demand-dynamic performance. *Comput. Chem. Eng.* **47**, 145–156 (2012).

[9] Suresh, P., Hsu, S.-H., Akkisetty, P., Reklaitis, G. V. & Venkatasubramanian, V. OntoMODEL: ontological mathematical modeling knowledge management in pharmaceutical product development, 1: conceptual framework. *Ind. Eng. Chem. Res.* **49**, 7758–7767 (2010).

[10] Wall, K. Complexity of chemical products, plants, processes and control systems. *Chem. Eng. Res. Des.* **87**, 1430–1437 (2009).

[11] Niederer, S. A., Sacks, M. S., Girolami, M. & Willcox, K. Scaling digital twins from the artisanal to the industrial. *Nat. Comput. Sci.* **1**, 313–320 (2021). 38217216 10.1038/s43588-021-00072-5

[12] Moser, A., Appl, C., Brüning, S. & Hass, V. C. in *Digital Twins: Tools and Concepts for Smart Biomanufacturing* (eds Herwig, C., Pörtner, R. & Möller, J.) 133–180 (Springer, 2021).

[13] Friederich, J., Francis, D. P., Lazarova-Molnar, S. & Mohamed, N. A framework for data-driven digital twins of smart manufacturing systems. *Comput. Ind.* **136**, 103586 (2022).

[14] Schweidtmann, A. M., Zhang, D. & von Stosch, M. A review and perspective on hybrid modeling methodologies. *Digit. Chem. Eng.* **10**, 100136 (2024).

[15] Agi, D. T. et al. Computational toolkits for model-based design and optimization. *Curr. Opin. Chem. Eng.* **43**, 100994 (2024).

[16] Delgado-Licona, F. & Abolhasani, M. Research acceleration in self-driving labs: technological roadmap toward accelerated materials and molecular discovery. *Adv. Intell. Syst.* **5**, 2200331 (2023).

[17] Hogan, A. et al. Knowledge graphs. *ACM Comput. Surv.* **54**, 71:1–71:37 (2021).

[18] Wilkinson, M. D. et al. The FAIR Guiding Principles for scientific data management and stewardship. *Sci. Data* **3**, 160018 (2016). 26978244 10.1038/sdata.2016.18 PMC4792175

[19] Pascazio, L. et al. Chemical species ontology for data integration and knowledge discovery. *J. Chem. Inf. Model.* **63**, 6569–6586 (2023). 37883649 10.1021/acs.jcim.3c00820 PMC10647085

[20] Garay-Ruiz, D. & Bo, C. Chemical reaction network knowledge graphs: the OntoRXN ontology. *J. Cheminform.* **14**, 29 (2022). 35637523 10.1186/s13321-022-00610-x PMC9153116

[21] Behr, A. S., Borgelt, H. & Kockmann, N. Ontologies4Cat: investigating the landscape of ontologies for catalysis research data management. *J. Cheminform.* **16**, 16 (2024). 38326906 10.1186/s13321-024-00807-2 PMC10851519

[22] Hailemariam, L. et al. in *Computer Aided Chemical**Engineering* (eds Braunschweig, B. & Joulia, X.) Vol. 25 85–90 (Elsevier, 2008).

[23] Yang, A. & Marquardt, W. in *Computer Aided Chemical**Engineering* (eds Barbosa-Póvoa, A. & Matos, H.) Vol. 18 1159–1164 (Elsevier, 2004).

[24] Kondinski, A., Bai, J., Mosbach, S., Akroyd, J. & Kraft, M. Knowledge engineering in chemistry: from expert systems to agents of creation. *Acc. Chem. Res.* **56**, 128–139 (2023). 36516456 10.1021/acs.accounts.2c00617 PMC9850921

[25] Baum, Z. J. et al. Artificial intelligence in chemistry: current trends and future directions. *J. Chem. Inf. Model.* **61**, 3197–3212 (2021). 34264069 10.1021/acs.jcim.1c00619

[26] Boiko, D. A., MacKnight, R., Kline, B. & Gomes, G. Autonomous chemical research with large language models. *Nature* **624**, 570–578 (2023). 38123806 10.1038/s41586-023-06792-0 PMC10733136

[27] Caprotti, O. & Carlisle, D. OpenMath and MathML: semantic markup for mathematics. *XRDS* **6**, 11–14 (1999).

[28] Morbach, J., Wiesner, A. & Marquardt, W. OntoCAPE—a (re)usable ontology for computer-aided process engineering. *Comput. Chem. Eng.* **33**, 1546–1556 (2009).

[29] Pérez, J., Arenas, M. & Gutierrez, C. Semantics and complexity of SPARQL. *ACM Trans. Database Syst.* **34**, 16:1–16:45 (2009).

[30] Sundar, K. P. & Kanmani, S. Progression of photocatalytic reactors and it’s comparison: a review. *Chem. Eng. Res. Des.* **154**, 135–150 (2020).

[31] Li, Q., Cheng, T., Wang, Y. & Bryant, S. H. PubChem as a public resource for drug discovery. *Drug Discov. Today* **15**, 1052–1057 (2010). 20970519 10.1016/j.drudis.2010.10.003 PMC3010383

[32] Pence, H. E. & Williams, A. ChemSpider: an online chemical information resource. *J. Chem. Educ.* **87**, 1123–1124 (2010).

[33] Ertl, P., Patiny, L., Sander, T., Rufener, C. & Zasso, M. Wikipedia Chemical Structure Explorer: substructure and similarity searching of molecules from Wikipedia. *J. Cheminform.* **7**, 10 (2015). 25815062 10.1186/s13321-015-0061-y PMC4374119

[34] Vermeire, F. H., Chung, Y. & Green, W. H. Predicting solubility limits of organic solutes for a wide range of solvents and temperatures. *J. Am. Chem. Soc.* **144**, 10785–10797 (2022). 35687887 10.1021/jacs.2c01768

[35] OpenAI et al. GPT-4 technical report. Preprint at https://arxiv.org/abs/2303.08774 (2024).

[36] Commenge, J.-M. & Falk, L. Villermaux–Dushman protocol for experimental characterization of micromixers. *Chem. Eng. Process. Process Intensif.* **50**, 979–990 (2011).

[37] Jose, N. A., Zeng, H. C. & Lapkin, A. A. Hydrodynamic assembly of two-dimensional layered double hydroxide nanostructures. *Nat. Commun.* **9**, 4913 (2018). 30464298 10.1038/s41467-018-07395-4 PMC6249219

[38] Baldyga, J. & Bourne, J. R. Simplification of micromixing calculations. I. Derivation and application of new model. *Chem. Eng. J.* **42**, 83–92 (1989).

[39] Reckamp, J. M. et al. Mixing performance evaluation for commercially available micromixers using Villermaux–Dushman reaction scheme with the interaction by exchange with the mean model. *Org. Process Res. Dev.* **21**, 816–820 (2017).

[40] Han, Y., Kanno, H., Ahn, Y.-J. & Shikazono, N. Measurement of liquid film thickness in micro tube annular flow. *Int. J. Multiph. Flow* **73**, 264–274 (2015).

[41] Fournier, M.-C., Falk, L. & Villermaux, J. A new parallel competing reaction system for assessing micromixing efficiency—Determination of micromixing time by a simple mixing model. *Chem. Eng. Sci.* **51**, 5187–5192 (1996).

[42] Falk, L. & Commenge, J.-M. Performance comparison of micromixers. *Chem. Eng. Sci.* **65**, 405–411 (2010).

[43] Zhang, F., Marre, S. & Erriguible, A. Mixing intensification under turbulent conditions in a high pressure microreactor. *Chem. Eng. J.* **382**, 122859 (2020).

[44] Schrimpf, M. et al. Taylor–Couette reactor: principles, design, and applications. *AIChE J* **67**, e17228 (2021).

[45] Nemri, M., Charton, S. & Climent, E. Mixing and axial dispersion in Taylor–Couette flows: the effect of the flow regime. *Chem. Eng. Sci.* **139**, 109–124 (2016).

[46] Tang, J., Wang, C., Liu, F., Yang, X. & Wang, R. Micromixing performance in a Taylor–Couette reactor with ribbed rotors. *Processes* **11**, 2058 (2023).

[47] Andereck, C. D., Liu, S. S. & Swinney, H. L. Flow regimes in a circular Couette system with independently rotating cylinders. *J. Fluid Mech.* **164**, 155–183 (1986).

[48] Tam, W. Y. & Swinney, H. L. Mass transport in turbulent Couette–Taylor flow. *Phys. Rev. A* **36**, 1374–1381 (1987). 10.1103/physreva.36.1374 9898995

[49] Moore, C. M. V. & Cooney, C. L. Axial dispersion in Taylor–Couette flow. *AIChE J* **41**, 723–727 (1995).

[50] Enokida, Y., Nakata, K. & Suzuki, A. Axial turbulent diffusion in fluid between rotating coaxial cylinders. *AIChE J* **35**, 1211–1214 (1989).

[51] Ohmura, N., Kataoka, K., Shibata, Y. & Makino, T. Effective mass diffusion over cell boundaries in a Taylor–Couette flow system. *Chem. Eng. Sci.* **52**, 1757–1765 (1997).

[52] Racina, A., Liu, Z. & Kind, M. in *Micro and Macro Mixing: Analysis, Simulation and Numerical Calculation* (eds Bockhorn, H., Mewes, D., Peukert, W. & Warnecke, H.-J.) 125–139 (Springer, 2010).

[53] Taylor, C. J. et al. A brief introduction to chemical reaction optimization. *Chem. Rev.* **123**, 3089–3126 (2023). 36820880 10.1021/acs.chemrev.2c00798 PMC10037254

[54] Jiscoot, N., A. Uslamin, E. & Pidko, A. E. Model-based evaluation and data requirements for parallel kinetic experimentation and data-driven reaction identification and optimization. *Digit. Discov.* **2**, 994–1005 (2023).

[55] Anastas, P. & Eghbali, N. Green chemistry: principles and practice. *Chem. Soc. Rev.* **39**, 301–312 (2009). 20023854 10.1039/b918763b

[56] Joseph, V. R., Gul, E. & Ba, S. Maximum projection designs for computer experiments. *Biometrika* **102**, 371–380 (2015).

[57] Villermaux, J. Micromixing phenomena in stirred reactors. *Encycl. Fluid Mech.* **2**, 707–771 (1986).

[58] Schikarski, T., Trzenschiok, H., Peukert, W. & Avila, M. Inflow boundary conditions determine T-mixer efficiency. *React. Chem. Eng.* **4**, 559–568 (2019).

[59] Li, G., Shang, M., Song, Y. & Su, Y. Characterization of liquid–liquid mass transfer performance in a capillary microreactor system. *AIChE J* **64**, 1106–1116 (2018).

[60] Frazier, P. I. A tutorial on Bayesian optimization. Preprint at https://arxiv.org/abs/1807.02811 (2018).

[61] Hone, C. A., Holmes, N., Akien, G. R., Bourne, R. A. & Muller, F. L. Rapid multistep kinetic model generation from transient flow data. *React. Chem. Eng.* **2**, 103–108 (2017). 28580177 10.1039/c6re00109b PMC5436494

[62] Lutze, P., Babi, D. K., Woodley, J. M. & Gani, R. Phenomena based methodology for process synthesis incorporating process intensification. *Ind. Eng. Chem. Res.* **52**, 7127–7144 (2013).

[63] Peschel, A., Freund, H. & Sundmacher, K. Methodology for the design of optimal chemical reactors based on the concept of elementary process functions. *Ind. Eng. Chem. Res.* **49**, 10535–10548 (2010).

[64] Lapkin, A. A., Voutchkova, A. & Anastas, P. A conceptual framework for description of complexity in intensive chemical processes. *Chem. Eng. Process. Process Intensif.* **50**, 1027–1034 (2011).

[65] Waldron, C. et al. Model-based design of transient flow experiments for the identification of kinetic parameters. *React. Chem. Eng.* **5**, 112–123 (2019).

[66] Glimm, B., Horrocks, I., Motik, B., Stoilos, G. & Wang, Z. HermiT: an OWL 2 reasoner. *J. Autom. Reason.* **53**, 245–269 (2014).

[67] Kockmann, N., Kiefer, T., Engler, M. & Woias, P. Convective mixing and chemical reactions in microchannels with high flow rates. *Sens. Actuators B* **117**, 495–508 (2006).

[68] Panić, S., Loebbecke, S., Tuercke, T., Antes, J. & Bošković, D. Experimental approaches to a better understanding of mixing performance of microfluidic devices. *Chem. Eng. J.* **101**, 409–419 (2004).

[69] Men, Y. et al. Determination of the segregation index to sense the mixing quality of pilot- and production-scale microstructured mixers. *Chem. Eng. Res. Des.* **85**, 605–611 (2007).

