# Supplementary material for: A knowledge graph framework for digital twins of chemical processes
Source: Nat Chem Eng. 2026 May 14;3(5):286–97. doi: 10.1038/s44286-026-00392-1 (PMC13212158; doi:10.1038/s44286-026-00392-1)
Supplement: Supplementary file 1 — Supplementary Triples 1 and 2, Figs. 1–16 and Tables 1–14. [file 44286_2026_392_MOESM1_ESM.pdf]

nature chemical engineering

Supplementary information https://doi.org/10.1038/s44286-026-00392-1

A knowledge graph framework for digital twins of chemical processes

In the format provided by the authors and unedited

Supplementary Information Table of Contents

1. Ontological representation
    a. Namespaces
    b. OntoModel and OntoProcess
    c. Custom model library
    d. Ontology customisation
2. Annular microreactor case
3. Ribbed TCR case
4. Amidation reaction in a flow system
5. Autonomous agent development
    a. (Parallel) Model assembly agent
    b. (Parallel) Model calibration agent
    c. Rule inference agent
    d. Database query agent and AI model agent
    e. LLM chat agent
6. Web deployment
7. Experiment methods

1. Ontological representation

We develop OntoModel and OntoProcess as manifolds for defining triples to model and process knowledge for chemical processes. We provide details of developed triples below.

a. Namespaces

Supplementary Triples S1 lists the namespaces included in OntoModel and OntoProcess. Several namespaces are included for general ontology practice. Some namespaces from OntoCAPE are included in OntoModel and OntoProcess for reusing and creating related entities.

OntoCAPE provides a comprehensive framework to represent chemical processes with multiple interconnected sub-domains. OntoModel and OntoProcess reuse `system`, `process_model`, `mathematical_model`, `SI_unit`, and `derived_SI_units` sub-domains. Some classes in these namespaces are reused in OntoModel and OntoProcess, as listed in Supplementary Table S1.

Supplementary Triples S1. Namespaces for OntoModel and OntoProcess.

```
<rdf:RDF xmlns:owl="http://www.w3.org/2002/07/owl#"
    xmlns:rdf="http://www.w3.org/1999/02/22-rdf-syntax-ns#"
    xmlns:xml="http://www.w3.org/XML/1998/namespace"
    xmlns:xsd="http://www.w3.org/2001/XMLSchema#"
    xmlns:rdfs="http://www.w3.org/2000/01/rdf-schema#"
    xmlns:terms="http://purl.org/dc/terms/"
    xmlns:system="file:/ontology/OntoCAPE/upper_level/system.owl#"
    xmlns:process_model="file:/ontology/OntoCAPE/model/
        process_model.owl#"
    xmlns:behavior="file:/ontology/OntoCAPE/chemical_process_system/
        CPS_behavior/behavior.owl"
    xmlns:mathematical_model="file:/ontology/OntoCAPE/model/
        mathematical_model.owl#"
    xmlns:SI_unit="file:/ontology/OntoCAPE/supporting_concepts/
        SI_unit/SI_unit.owl#"
    xmlns:derived_SI_units="file:/ontology/OntoCAPE/
        supporting_concepts/SI_unit/derived_SI_units.owl#">
    …
</rdf:RDF>
```

Supplementary Table S1. Reused classes and corresponding namespaces from OntoCAPE.

**Class**	**Namespace**
`PhysicalDimension`	`system`
`UnitOfMeasure`	`system`
`Law`	`process_model`
`ProcessModel`	`process_model`
`Accumulation`	`behavior`
`FlowPattern`	`behavior`
`MolecularTransportPhenomenon`	`behavior`
`ChemicalReactionPhenomenon`	`behavior`
`Constant`	`mathematical_model`
`MathematicalModel`	`mathematical_model`
`ModelVariable`	`mathematical_model`
`Parameter`	`mathematical_model`
`Variable`	`mathematical_model`
`SI_BaseUnit`	`SI_unit`
`SI_DerivedBaseUnit`	`SI_unit`
`isDefinedBy`	`drived_SI_units`

b. OntoModel and OntoProcess

We develop OntoModel and OntoProcess to represent model and process knowledge in the realm of chemical engineering. OntoModel and OntoProcess include classes extended from OntoCAPE[1] or some basic ontologies, as listed in Supplementary Table S2. The extended relationships are also given in Supplementary Table S3. A virtualisation of the knowledge graph, which has been embedded with knowledge related to cases in this work, is shown as a topological graph in Supplementary Fig. S1.

Supplementary Table S2. Classes and corresponding parent OntoCAPE classes in OntoModel.

Class	Parent class
Variable (OntoModel)	ModelVariable (OntoCAPE)
Descriptor (OntoProcess)	NonExhaustiveValueSet (fundamental_concepts)
StructureDescriptor (OntoProcess)	Descriptor (OntoProcess)
SpaceDescriptor (OntoProcess)	Descriptor (OntoProcess)
EnergyDescriptor (OntoProcess)	Descriptor (OntoProcess)
SubstanceDescriptor (OntoProcess)	Descriptor (OntoProcess)
InformationDescriptor (OntoProcess)	Descriptor (OntoProcess)
TimeDescriptor (OntoProcess)	Descriptor (OntoProcess)
DataSource (OntoModel)	NonExhaustiveValueSet (fundamental_concepts)
Definition (OntoModel)	ProcessModel (OntoCAPE)
ModelDimension	PhysicalDimension (OntoCAPE)
Rule	NonExhaustiveValueSet (fundamental_concepts)
Context	NonExhaustiveValueSet (fundamental_concepts)

Supplementary Table S3. Relationships of OntoModel and OntoProcess, as well as corresponding parent relationships.

Relationship	Parent relationship
hasDefinition (OntoModel)	hasProperty (OntoCAPE)
hasDifferentialInitialValue (OntoModel)	hasProperty (OntoCAPE)
hasDifferentialModelVariable (OntoModel)	hasProperty (OntoCAPE)
hasDifferentialUpperLimit (OntoModel)	hasProperty (OntoCAPE)
hasLaw (OntoModel)	hasProperty (OntoCAPE)
hasOptionalModelVariable (OntoModel)	hasProperty (OntoCAPE)
hasRule (OntoModel)	hasProperty (OntoCAPE)
relatesToFlowPattern (OntoModel)	object-featureRelation (fundamental_concepts)
relatesToMolecularTransportPhenomenon (OntoModel)	behavior (OntoCAPE)
hasDescriptor (OntoProcess)	hasProperty (OntoCAPE)
hasStandardUnitOfMeasure (OntoModel, OntoProcess)	object-featureRelation (fundamental_concepts)

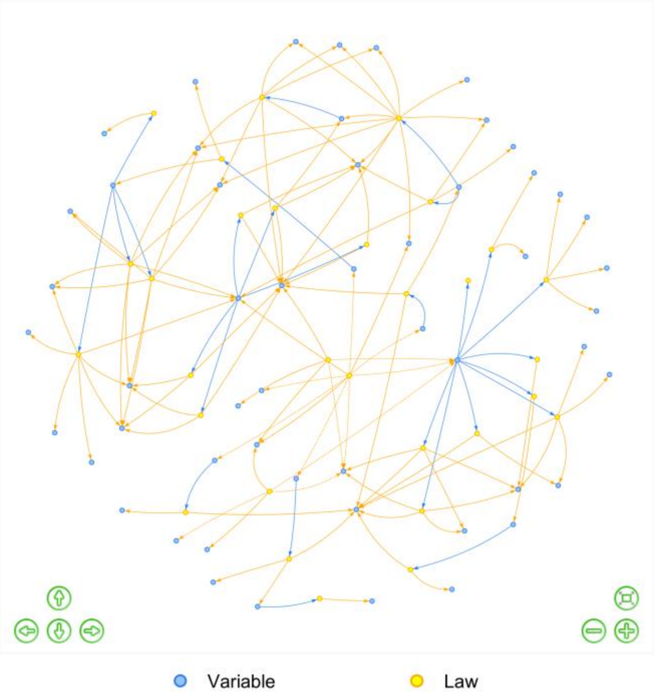

Supplementary Fig. S1 Visualisation of law and variable nodes related to cases of this work in a partial knowledge graph.

c. Custom model library

A customised model library has been developed in the study (as listed in Supplementary Table S4) to include the most widely used equations, including mass balance equations and kinetics equations, to support model construction.

Supplementary Table S4 An example model library in OntoModel.

Model	Description	MathML[2]
Plug Flow	The fluid's velocity is constant across any cross-section of the pipe.	<mfrac><br><mn>1</mn><br><mi>u</mi><br></mfrac><br><mrow><br><mo>(</mo><br><mrow><br><mo>matmul</mo><br><msub><br><mi>r</mi><br><mtext>r</mtext><br></msub><br><mi>v</mi><br></mrow><br><mo>)</mo><br></mrow>
Batch	The reactor volume is a constant when used to transform reactants into products.	<mrow><br><mo>matmul</mo><br><msub><br><mi>r</mi><br><mtext>r</mtext>

		</msub><br><mi>v</mi><br></mrow>
Continuous Stirred Tank	Large tanks with stirring mechanisms that keep reactants evenly distributed.	<msub><br><mi>c</mi><br><mtext>0</mtext><br></msub><br><mo>+</mo><br><mfrac><br><mi>V</mi><br><mi>q</mi><br></mfrac><br><mrow><br><mo>matmul</mo><br><msub><br><mi>r</mi><br><mtext>r</mtext><br></msub><br><mi>v</mi><br></mrow>
Plain Rate Constant	Reaction rate constant is a plain number.	<mi>k</mi>
Concentration Power Dependence	Reaction rate increases as concentration to some power.	<mrow><br><mo>∏</mo><br><msup><br><mi>c</mi><br><mi>n</mi><br></msup><br></mrow>
Photocatalysis	A type of catalysis that results in the modification of the rate of a photoreaction.	<mfrac><br><mrow><br><mi>α</mi><br><mi>I</mi><br></mrow><br><mrow><br><mn>1</mn><br><mo>+</mo><br><mi>α</mi><br><mi>I</mi><br></mrow><br></mfrac>
Langmuir-Hinshelwood	A reaction that occurs on a catalyst surface, where reactants adsorb onto the surface and interact to form products.	<mfrac><br><mrow><br><mi>k</mi><br><msup><br><msub><br><mi>W</mi><br><mtext>cat</mtext><br></msub><br><mn>2</mn><br></msup><br><mrow><br><mo>∏</mo><br><mrow><br><mi>c</mi><br><mo>[</mo><br><mi>v</mi><br><mo><</mo><br><mn>0</mn><br><mo>]</mo><br></mrow><br></mrow><br></mrow><br><mrow><br><msup><br><mrow><br><mo>(</mo><br><mn>1</mn><br><mo>+</mo><br><mrow>

		<mo>∑</mo><br><mrow><br><mi>K</mi><br><mi>c</mi><br></mrow><br></mrow><br><mo>)</mo><br></mrow><br><mn>2</mn><br></msup><br></mrow><br></mfrac>
Eley-Rideal	A reaction that occurs when one reactant is adsorbed on the catalyst surface and other reactants are in the gas/liquid phase.	<mfrac><br><mrow><br><mi>k</mi><br><msub><br><mi>W</mi><br><mtext>cat</mtext><br></msub><br><mrow><br><mo>∏</mo><br><mrow><br><mi>c</mi><br><mo>[</mo><br><mi>v</mi><br><mo><</mo><br><mn>0</mn><br><mo>]</mo><br></mrow><br></mrow><br></mrow><br><mrow><br><mn>1</mn><br><mo>+</mo><br><mrow><br><mo>∑</mo><br><mrow><br><mi>K</mi><br><mi>c</mi><br></mrow><br></mrow><br></mrow><br></mfrac>
Diffusion	Molecules move from regions of higher concentration to regions of lower concentration due to random thermal motion.	<mrow><br><msub><br><mi>K</mi><br><mtext>c</mtext><br></msub><br><mi>Δc</mi><br></mrow>
Engulfment	The formation and dissipation of vortices lead to a homogenisation process of concentration.	<mfrac><br><mrow><br><msub><br><mi>c</mi><br><mtext>m</mtext><br></msub><br><mo>−</mo><br><mi>c</mi><br></mrow><br><msub><br><mi>t</mi><br><mtext>m</mtext><br></msub><br></mfrac>
Liquid-liquid Mass Transfer	The process where a solute moves between two immiscible liquid phases due to concentration gradients.	<mrow><br><mrow><br><msub><br><mi>k</mi><br><mtext>L</mtext>

```
                    </msub>
                    <mi>a</mi>
                </mrow>
                <mi>Δc</mi>
            </mrow>
```

d. Ontology customisation

We provide a workflow to customise the OWL (Web Ontology Language) file by adding new `Variables` and `Laws`, as well as associated instances of `Phenomenon`, `Unit`, `Descriptor`, and `Rule`, via SPARQL update queries (Supplementary Fig. S2). JSON is adopted for structuring these new instances into a machine-readable format that can be automatically parsed and translated into SPARQL update queries. Example JSON files can be found at https://github.com/sustainable-processes/KG4DT/tree/main/graphdb/ontology/patches for adding `Laws` of mixing rate for diffusion, reaction rate for Eley-Rideal surface catalysis, and reaction rate for Arrhenius referenced to 293 K.

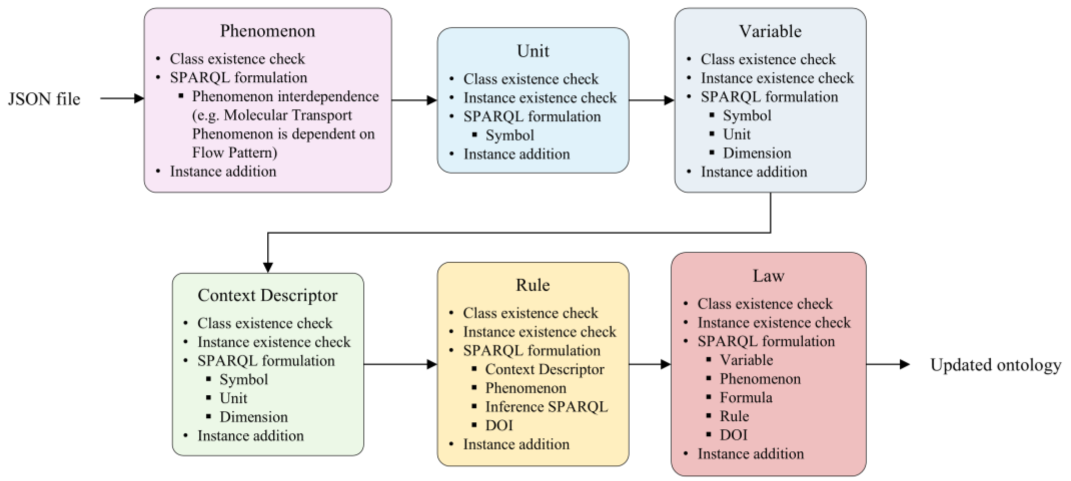

Supplementary Fig. S2 Illustration of the workflow for model ontology customisation.

2. Annular microreactor

The Villermaux-Dushman reaction system[3] is operated in a custom-built annular microreactor, as shown in Supplementary Fig. S3, as a bottom-up model construction demonstration, including the following reactions:

$$H_2BO_3^- + H^+ \rightarrow H_3BO_3 \quad (1)$$

$$5I^- + IO_3^- + 6H^+ \rightarrow 3I_2 + 3H_2O \quad (2)$$

$$I_2 + I^- \rightleftharpoons I_3^- \quad (3)$$

Reaction (1) is a quasi-instantaneous reaction, and its reaction rate depends on the

streaming mixing rate. Once H^+ enters the stream volume with dissolved $H_2BO_3^-$, it will be immediately consumed by this neutralisation reaction. Reaction (2) is also fast, and its kinetics has been reported as $r_2 = k_2[I^-]^2[IO_3^-][H^+]^2$, where k_2 is dependent on the ionicity I. When $I < 0.16$ M, log k_2 is given as $9.28 - 3.66I^{0.5}$; otherwise, log k_2 is given as $8.38 - 1.51I^{0.5} + 0.23I$. For reaction (3), the partial order is 1 for reactants in both forward and backward reactions. The rate constant is 5.6×10^9 M^{-1} s^{-1} for the forward reaction and 7.6×10^6 s^{-1} for the backward reaction. Supplementary Tables S5 and S6 list our operation conditions and experimental data in the annular microreactor, respectively. Supplementary Table S7 provides details of the referenced T-mixer[4], IMTEK[5], and IMM[6]. The database query agent is designed to send requests to all registered databases and parse returned contents to extract the physicochemical properties of used solvents (Supplementary Fig. S4). Similarly, the AI model agent would also invoke the configured application programming interface (API) for predicting solubilities of species in the Villermaux-Dushman reaction system.

Supplementary Table S5. Operation conditions of the annular microreactor.

Re	Phase Ratio q_G/q_L	Residence Time (s)
5.0–306.1	10–750	0.014–1.146

Supplementary Table S6. Experimental data of the annular microreactor case.

Flow Rate			Outlet Concentration
q_{L1} (mL min^{-1})	q_{L2} (mL min^{-1})	q_G (L min^{-1})	$[I_3^-]$ (M)
0.2	0.2	0.2	0.002025
0.4	0.4	0.2	0.001813
0.6	0.6	0.2	0.001692
0.8	0.8	0.2	0.001574
1.0	1.0	0.2	0.001429
0.2	0.2	0.3	0.001789
2.0	2.0	0.2	0.001292
0.6	0.6	0.3	0.001528
0.8	0.8	0.3	0.001449
4.0	4.0	0.2	0.001207
6.0	6.0	0.2	0.001165
8.0	8.0	0.2	0.001045
10.0	10.0	0.2	0.000906
10.0	10.0	0.3	0.000943
12.0	12.0	0.8	0.000304
4.0	4.0	1.0	0.000355

6.0	6.0	1.0	0.000402
10.0	10.0	1.25	0.000203
10.0	10.0	1.5	0.000152
6.0	6.0	2.0	0.000166
10.0	10.0	1.75	0.000207
10.0	10.0	2.25	0.000118
10.0	10.0	2.5	0.000091
10.0	10.0	2.75	0.000101

Supplementary Table S7. Details of referenced mixers.

Mixer	Description	Observation
T-mixer	A T-mixer produced by Bohlender GmbH, Grünsfeld, Germany, with an internal channel diameter of approximately 800 µm.	based on split-and-recombine principle
IMTEK	A triangular interdigital micromixer invented by Mgt Mikroglas Technik, of which the fluid feed channels are 50 µm in width and 150 µm in height.	based on multi-lamination principle
IMM	The IMM catalogue commercialised by Institut für Mikrotechnik Mainz GmbH, Germany, with several tens, hundreds, or thousands of stacked star-shaped foils as mixing elements. These stacked foils form hundreds to thousands of micro-plumes into the cylindrical mixing flow-through chamber (Ø 5.6 mm × 16.8 mm).	based on multi-lamination principle

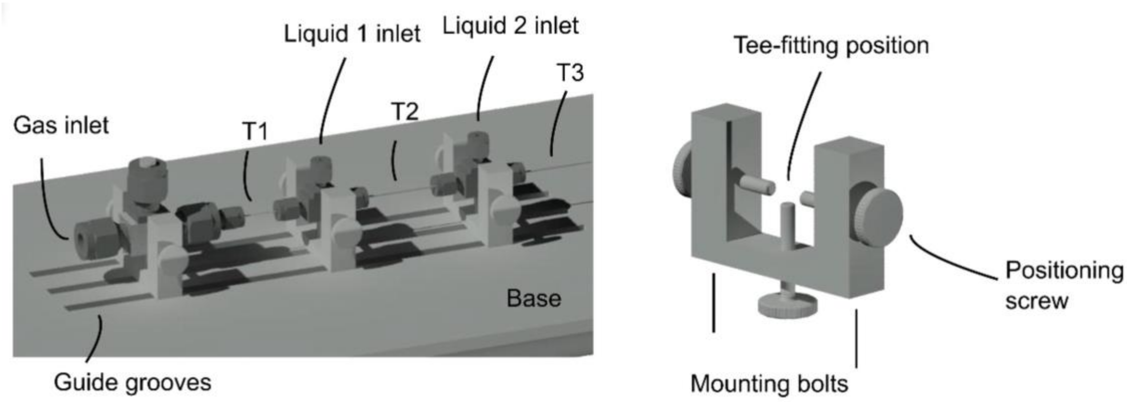

Supplementary Fig. S3 Custom-built device for annular flow experiments. Three quartz tubes were arranged in a tube-in-tube configuration. In T2, the injected air stream forces Liquid 1 into a thin film, which then contacts with a thin film of Liquid 2 in T3

for mixing and reaction. Stainless steel tee connectors with 1/4” and 1/16” compression fittings were obtained from Swagelok.

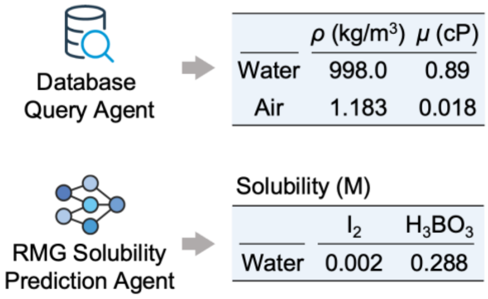

Supplementary Fig. S4 Illustration of physicochemical property acquirement by the autonomous database query and AI model agents.

3. Ribbed TCR case

Supplementary Fig. S5 shows the structure of the ribbed Taylor-Couette reactor (TCR, from Autichem Ltd.). It consists of a glass cylinder outside with an effective length of 20 cm, and a rotating rotor inside made of ceramic and attached with ribs. The rotor is controlled by a motor with adjustable speed ranging from 0 to 360 rpm. Three operating scenarios with different rotating speeds or solvents are applied to demonstrate the top-down model search approach. The initial concentrations of 2-bromobenzyl alcohol (BBA) and acetic anhydride (Ac_2O) are set to 0.20 M. Supplementary Table S8 lists SPARQL codes used in the applicability evaluation for each correction of the axial dispersion coefficient. The experimental data are listed in Supplementary Table S9. The calibration results are given in Supplementary Table S10 for the top-down searched models under each operating condition scenario.

The synthesis procedure, HPLC calibration curves, and NMR spectra for analysis of reaction results are provided below. We prepared 2-bromobenzyl acetate (BBAc) as follows: Ac_2O (2.0 equiv.) was added to a solution of BBA (1.0 equiv.), Et_3N (2.0 equiv.), and DMAP (0.10 equiv.) in EtOAc (0.20 M) at ambient conditions. After the solution was stirred at the same temperature for 30 min, the resultant mixture was concentrated in *vacuo* at room temperature. The crude product was purified by column chromatography (EtOAc/hexane = 1/4).

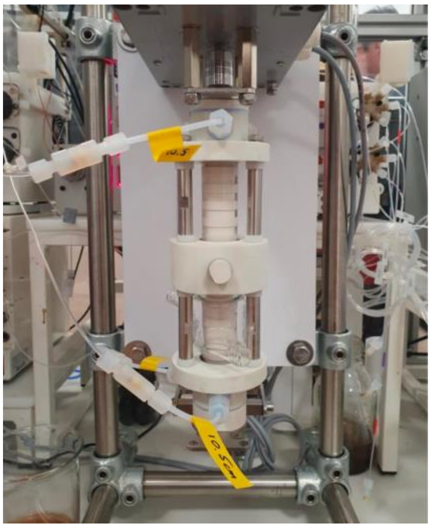

Supplementary Fig. S5 Photograph of the Ribbed TCR.

Supplementary Table S8. SPARQL codes for evaluating applicabilities of D_x corrections.

<table>
<tr><th>Authors</th><th>Flow pattern</th><th>SPARQL codes</th></tr>
<tr><td>Ohmura et al.</td><td>Vortex Flow</td><td>select ?is_rule_match where {
    bind({Taylor-Couette_Reactor} as ?tcr).
    bind({Has_Gas_Phase} as ?gas).
    bind({Is_Liquid_Phase_Miscible} as ?mis).
    bind({Reactor_Inner_Radius} as ?ri).
    bind({Reactor_Outer_Radius} as ?ro).
    bind({Phase_Density} as ?rho).
    bind({Phase_Viscosity} as ?mu).
    bind({Rotational_Angular_Velocity} as ?ω).
    bind(
        if(
            ?tcr && !?gas && ?mis &&
            ((?ro-?ri = 0.00184) ||
            (?ri/?ro = 0.7) ||
            ((?ro-?ri)*?ω*?ri*?rho/?mu > 1200 &&
            (?ro-?ri)*?ω*?ri*?rho/?mu < 1700)),
            true,
            false
) as ?is_rule_match
).
}</td></tr>
</table>

Ohmura et al. | Turbulent Flow

```
select ?is_rule_match where {
    bind({Taylor-Couette_Reactor} as ?tcr).
    bind({Has_Gas_Phase} as ?gas).
    bind({Is_Liquid_Phase_Miscible} as ?mis).
    bind({Reactor_Inner_Radius} as ?ri).
    bind({Reactor_Outer_Radius} as ?ro).
    bind({Phase_Density} as ?rho).
    bind({Phase_Viscosity} as ?mu).
    bind({Rotational_Angular_Velocity} as ?ω).
    bind(
        if(
            ?tcr && !?gas && ?mis &&
            ((?ro-?ri = 0.00184) ||
            (?ri/?ro = 0.7) ||
            (?ro-?ri)*?ω*?ri*?rho/?mu > 1700)),
            true,
            false
        ) as ?is_rule_match
    ).
}
```

Tam and Swinney | Turbulent Flow

```
select ?is_rule_match where {
    bind({Taylor-Couette_Reactor} as ?tcr).
    bind({Has_Gas_Phase} as ?gas).
    bind({Is_Liquid_Phase_Miscible} as ?mis).
    bind({Reactor_Inner_Radius} as ?ri).
    bind({Reactor_Outer_Radius} as ?ro).
    bind({Phase_Density} as ?rho).
    bind({Phase_Viscosity} as ?mu).
    bind({Rotational_Angular_Velocity} as ?ω).
    bind(
        if(
            ?tcr && !?gas && ?mis &&
            (((?ro-?ri > 0.0032 && ?ro-?ri < 0.01285) ||
            (?ri/?ro > 0.494 && ?ri/?ro < 0.875)) ||
            ((?ro-?ri)*?ω*?ri*?rho/?mu > 3600)),
            true,
            false) as ?is_rule_match
    ).
}
```

Racina et al. | Turbulent Flow

```
select ?is_rule_match where {
    bind({Taylor-Couette_Reactor} as ?tcr).
    bind({Has_Gas_Phase} as ?gas).
    bind({Is_Liquid_Phase_Miscible} as ?mis).
    bind({Reactor_Inner_Radius} as ?ri).
    bind({Reactor_Outer_Radius} as ?ro).
    bind({Phase_Density} as ?rho).
    bind({Phase_Viscosity} as ?mu).
    bind({Rotational_Angular_Velocity} as ?ω).
    bind(
        if(
            ?tcr && !?gas && ?mis &&
            (((?ro-?ri > 0.012 && ?ro-?ri < 0.0185) ||
            (?ri/?ro > 0.68 && ?ri/?ro < 0.76)) ||
            ((?ro-?ri)*?ω*?ri*?rho/?mu > 800)),
            true,
            false) as ?is_rule_match
    ).
}
```

Enokida et al. | Taylor-Couette Flow

```
select ?is_rule_match where {
    bind({Taylor-Couette_Reactor} as ?tcr).
    bind({Has_Gas_Phase} as ?gas).
    bind({Is_Liquid_Phase_Miscible} as ?mis).
    bind({Reactor_Inner_Radius} as ?ri).
    bind({Reactor_Outer_Radius} as ?ro).
    bind({Phase_Density} as ?rho).
    bind({Phase_Viscosity} as ?mu).
    bind({Rotational_Angular_Velocity} as ?ω).
    bind(
        if(
            ?tcr && !?gas && ?mis &&
            (((?ro-?ri > 0.018 && ?ro - ?ri < 0.0305) ||
            (?ri/?ro > 0.59 && ?ri/?ro < 0.76)) ||
            ((?ro-?ri)*?ω*?ri*?rho / ?mu > 0)),
            true,
            false) as ?is_rule_match
    ).
}
```

Moore and Conney | Taylor-Couette Flow

```
select ?is_rule_match where {
    bind({Taylor-Couette_Reactor} as ?tcr).
    bind({Has_Gas_Phase} as ?gas).
    bind({Is_Liquid_Phase_Miscible} as ?mis).
    bind({Reactor_Inner_Radius} as ?ri).
    bind({Reactor_Outer_Radius} as ?ro).
    bind({Phase_Density} as ?rho).
    bind({Phase_Viscosity} as ?mu).
    bind({Rotational_Angular_Velocity} as ?ω).
    bind(
        if(
            ?tcr && !?gas && ?mis &&
            (((?ro-?ri > 0.0004 && ?ro-?ri < 0.0038) ||
            (?ri/?ro > 0.73 && ?ri/?ro < 0.96)) ||
            ((?ro-?ri)*?ω*?ri*?rho/?mu > 100)),
            true,
            false) as ?is_rule_match
    ).
}
```

Degenerated | Taylor-Couette Flow

```
select ?is_rule_match where {
    bind({Taylor-Couette_Reactor} as ?tcr).
    bind({Has_Gas_Phase} as ?gas).
    bind({Is_Liquid_Phase_Miscible} as ?mis).
    bind(
        if(?tcr && !?gas && ?mis, true, false)
        as ?is_rule_match
    ).
}
```

Supplementary Table S9. Experiment results of BBAc concentration (M) at the outlet of the TCR under different operating conditions.

**Flow Rate *q* (mL min^{-1})**	**Base Scenario MeCN 360 rpm**	**60 rpm**	**Varied *ω* Scenario MeCN 0 rpm**	**Varied Solvent Scenario Toluene 360 rpm**
8.50	0.0247	0.0271	0.0182	0.0220
4.25	0.0383	0.0398	0.0337	0.0321
2.13	0.0530	0.0567	0.0412	0.0444
1.42	0.0627	0.0647	0.0475	0.0539
1.00	0.0693	0.0715	0.0540	0.0622

0.85	-	-	0.0570	0.0648

Supplementary Table S10. Calibration results of the top-down searched models of the ribbed TCR under different operating conditions.

**Base Scenario**<br>**MeCN**<br>**60 or 360 rpm**	**Varied ω Scenario**<br>**MeCN**<br>**0 rpm**	**Varied Solvent Scenario**<br>**EtOAc**<br>**60 rpm**
Moore and Conney	Constant D_x	Moore and Conney
$k_{D_x} = 3.58 \times 10^{-9}\ \mathrm{m^2\ s^{-1}}$	$D_{\mathrm{const}} = 9.98 \times 10^{-4}\ \mathrm{m^2\ s^{-1}}$	$k_{D_x} = 1.34 \times 10^{-8}\ \mathrm{m^2\ s^{-1}}$
$k = 0.051\ \mathrm{M^{-1}s^{-1}}$	$k = 0.051\ \mathrm{M^{-1}s^{-1}}$	$k = 0.042\ \mathrm{M^{-1}s^{-1}}$

4. Amidation reaction in a flow system

An amidation reaction of benzylamine (BA) and acetic anhydride (Ac_2O) is performed in a T-mixer to verify the application of our knowledge graph framework in multi-objective optimisation in this case, targeting at the production of N-benzylacetamide (BAA). The main and side reactions are as follows:

$$\text{Main: BA} + Ac_2O \rightarrow \text{BAA} + CH_3COOH \quad (4)$$

$$\text{Side: } Ac_2O + H_2O \rightarrow 2CH_3COOH \quad (5)$$

A T-mixer is used as the reactor with MeCN and EtOAc as organic solvent options. The LLM chat agent is queried for investigating possible molecular transport phenomena and associated formulas for a T-mixer (Supplementary Fig. S6). The database query agent results reveal that MeCN is miscible with water to form a single-phase system; EtOAc is immiscible and forms a two-phase system with water. Variables and their ranges for optimising the amidation reaction are listed in Supplementary Table S11. Some experiments are conducted to collect data as the initial points for calibrating models. The initial points are summarised in Supplementary Table S12. Electrophile and NaOH are of the same concentration in the experiments. K_c is initialised with the inverse of the diffusion time, which can be written as d^2/D. Experimental points are sampled along the overall Pareto front to verify the effectiveness of constructed physical models in reaction optimisation (Supplementary Fig. S7). The calibration results of identified models are given in Supplementary Table S13. Supplementary Table S14 gives the final Pareto front explored by experiments. The physical model-based method is demonstrated to substantially outperform Bayesian optimisation[7], which adopts the Expected Improvement acquisition function, on a virtual nucleophilic aromatic substitution benchmark for maximising the target product yield, as shown in

Supplementary Fig. S8.

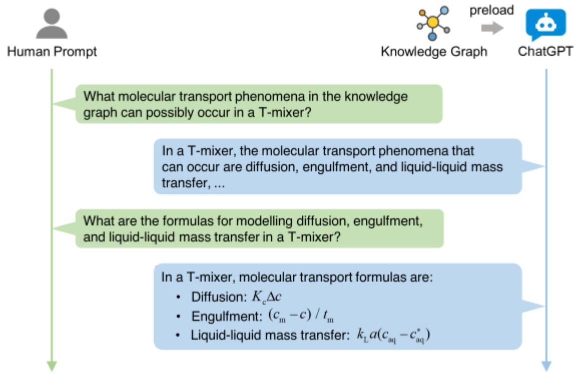

Supplementary Fig. S6 Human-LLM communication for querying possible molecular transport phenomena in a T-mixer.

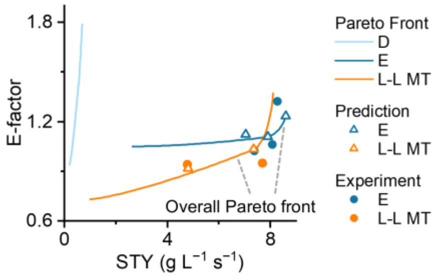

Supplementary Fig. S7 Predicted and experimental results of sample points for the physical model. Sampled points are distributed along the overall Pareto front to verify the optimised trade-off between STY and E-factor under both engulfment and liquid-liquid mass transfer mechanisms. D: diffusion; E: engulfment; L-L MT: liquid-liquid mass transfer.

a

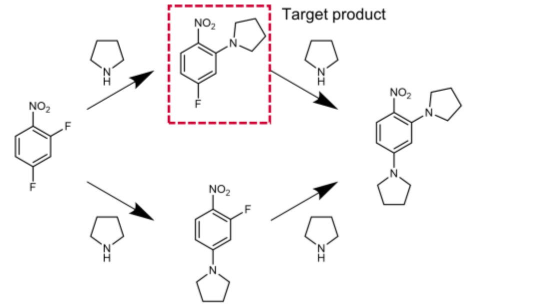

The substrate 2,4-difluoro-1-nitrobenzene has an inlet concentration of 0.1 M.

Operation parameter	Lower bound	Upper bound
Pyrroline equiv.	1	5
Residence time (min)	0.5	2
Temperature (°C)	30	120

b

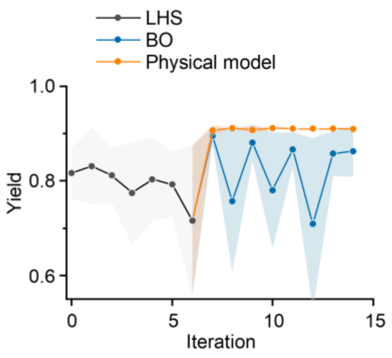

Supplementary Fig. S8 Benchmark of physical model-based reaction optimisation on a virtual nucleophilic aromatic substitution (S_NAr) reaction system performed in flow. **a**, The S_NAr reaction scheme and the experimental design region. **b**, Comparison of physical model-based method against Bayesian optimisation (BO) using the Expected Improvement acquisition function on the task of yield maximisation. The results are obtained from 8 independent runs with different random seeds. For each run, 7 experimental points are selected from the design space using Latin Hypercube Sampling[8], followed by 8 experimental points suggested by BO or the fitted physical model.

Supplementary Table S11. Variable ranges of the amidation reaction optimisation.

Variable	Range	Unit
Organic solvent	MeCN or EtOAc	-
Ac_2O equiv.	1.0–1.5	-
Total flow rate	0.2–12.0	mL min^{-1}

Supplementary Table S12. Initial experiment points of the amidation reaction optimisation case.

**Organic solvent**	**Total flow rate (mL min^{-1})**	**Ac_2O concentration (M)**	**Yield (%)**	**STY (g L^{-1} s^{-1})**	***E*-factor**
MeCN	0.20	0.39	97.6	0.15	1.00
MeCN	1.00	0.39	76.4	0.57	1.56
MeCN	4.00	0.39	92.2	2.75	1.12
MeCN	8.00	0.39	91.8	5.48	1.13
MeCN	10.24	0.39	90.8	6.94	1.15
MeCN	12.00	0.39	92.4	8.27	1.12
EtOAc	0.20	0.39	99.0	0.15	0.98
EtOAc	1.00	0.39	98.0	0.73	1.00
EtOAc	4.00	0.39	96.3	2.87	1.03
EtOAc	8.00	0.39	94.7	5.65	1.07
EtOAc	11.60	0.39	90.0	7.79	1.17
EtOAc	12.00	0.39	89.4	8.00	1.19
EtOAc	2.68	0.42	98.2	1.96	1.09
EtOAc	10.76	0.45	93.2	7.48	1.30

Supplementary Table S13. Calibration results of identified models in the amidation reaction optimisation case. k_{main} and k_{side} denote rate constants of the main and side reactions, respectively. Partition coefficient is derived from the RMG solubility prediction model results.

MeCN	EtOAc
$k_{main} = 20.25\ M^{-1}\ s^{-1}$	$k_{main} = 19.79\ M^{-1}\ s^{-1}$
$k_{side} = 1.30\times10^{-2}\ M^{-1}\ s^{-1}$	$k_{side} = 1.30\times10^{-2}\ M^{-1}\ s^{-1}$
$K_{c,Ac_2O} = 3.32\times10^{-2}\ s^{-1}$	$k_La = 6.98\times10^{-2}\ s^{-1}$
$K_{c,BA} = 1.01\times10^{-2}\ s^{-1}$	$P_{Ac_2O} = 20.26$
$f_m = 2.12$	$P_{BA} = 5.95$

Supplementary Table S14. The experimentally explored Pareto front of the amidation reaction optimisation.

Method	Organic solvent	Total flow Rate (mL min^{-1})	Ac_2O concentration (M)	Yield (%)	STY (g L^{-1} s^{-1})	*E*-factor
Physical model	EtOAc	7.04	0.33	90.9	4.78	0.94
	EtOAc	12.00	0.30	85.9	7.69	0.95
	MeCN	12.00	0.36	90.3	8.09	1.06
	MeCN	12.00	0.39	92.4	8.27	1.12
	MeCN	12.00	0.45	92.6	8.29	1.32
Bayesian optimisation	EtOAc	0.20	0.39	99.0	0.15	0.98
	MeCN	12.00	0.30	82.6	7.39	1.02
	MeCN	12.00	0.39	92.4	8.27	1.12
	MeCN	12.00	0.45	92.6	8.29	1.32

5. Autonomous agent development

Autonomous functional agents are incorporated into OntoProcess and OntoModel for providing facile handlers to the user to effectively acquire the target formatted knowledge. The lifecycle of the physical model, including the formulation, calibration, operation, and maintenance, is supposed to be supported by autonomous agents to complete the detailed steps while leaving the high-level operation to humans. We implement agents for (parallel) model assembly, (parallel) model calibration, rule inference, database query, AI model, and LLM chat. It is noted that more agents can be designed and developed to achieve various deliverables for realising the digital twin technology for chemical processes.

a. (Parallel) Model assembly agent

Model assembly is realised as the most fundamental and core function in the developed framework, functioning as an assembly line that operates according to provided real-world phenomena or specified `Laws`, analogous to a real line in a factory, but the products are models used as chemical process digital twins.

In the top-down search approach, multiple model candidates are proposed to be evaluated. Therefore, the assembly agent is parallelised by using multi-processing programming for acceleration. The (parallel) assembly agent is shown in Supplementary Fig. S9. MathML is parsed into an element tree and then the target language, e.g., Python. The conversion process is realised by a nested function, which parses the element tree from top to bottom.

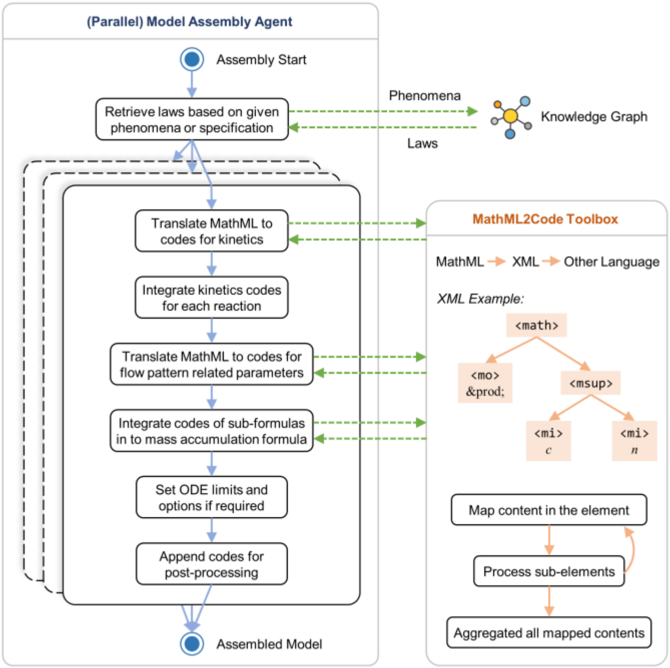

Supplementary Fig. S9 Flowsheet of the (parallel) model assembly agent. Laws are retrieved from the knowledge graph with given phenomena. For each law, the MathML formula is transformed to other languages using a MathML2Code toolbox. MathML is parsed into an element tree in the Extensible Markup Language (XML) and mapped to the target language for aggregation.

b. (Parallel) Model calibration agent

Model calibration is to align the internal function of the model with the real-world data by optimising related parameters. It is a crucial step for accommodating models in practical scenarios. This calibration agent should be flexible to varying fitting parameters and boundaries while being robust enough to be used in different calibration

tasks. In this way, some calibrated sub-modules of a digital twin can be directly reused. That is, for instance, reaction kinetics should be the same in different reactors if chemicals remain the same, which has been widely taken as a knowledge consensus.

We adopt a heuristics algorithm, differential evolution, to optimise assigned parameters with given boundaries. Root mean squared errors (RMSEs) of concentration are calculated and summed together as the minimisation target. The calibration flowsheet is provided in Supplementary Fig. S10.

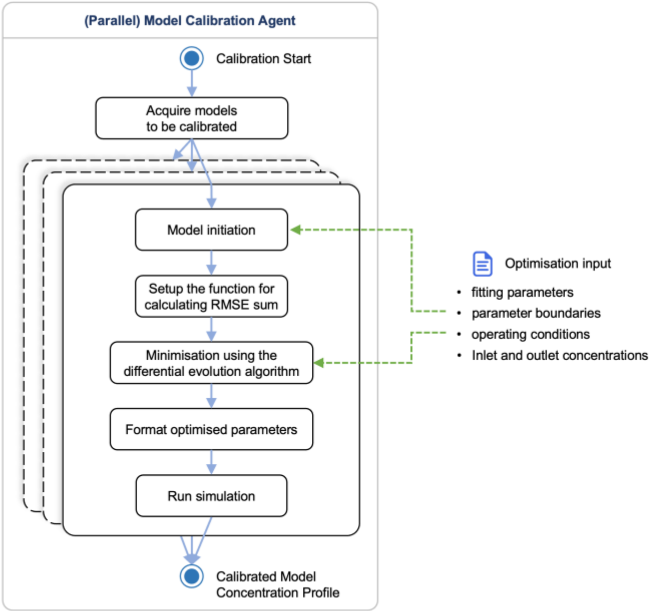

Supplementary Fig. S10 Flowsheet of the (parallel) model calibration agent. Minimising the summed RMSE is set as the calibration target. After calibration, a simulation is conducted to provide the concentration profile along with the calibrated model as the output.

c. Rule inference agent

In the top-down model search approach, the applicability of a law in certain operating conditions is determined by the result of defined SPARQL[9] codes. Supplementary Fig. S11 illustrates the rule inference procedure. Descriptors are set to detailed values, and then the SPARQL codes output a Boolean value as a result of the judgement statements. Laws that match the process context with at least one decisive descriptor are all used in constructing candidate models to search applicable models to the maximum extent.

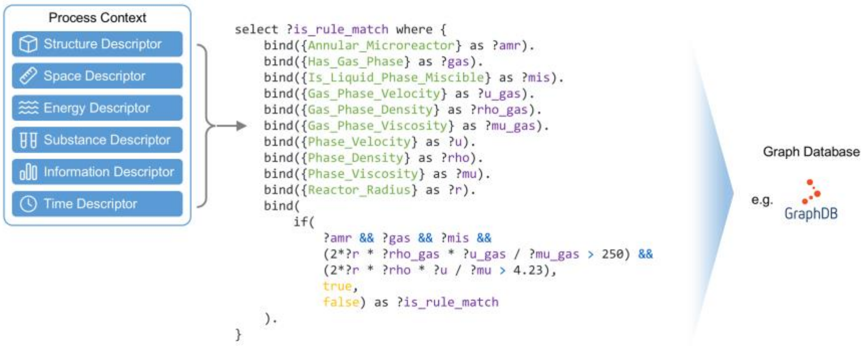

Supplementary Fig. S11 Illustration of rules inference by running SPARQL codes in a graph database engine (e.g., Graph DB) for the annular microreactor.

d. Database query agent and AI model agent

The database query and AI model agents are utilised with the provided APIs, which are managed in the knowledge graph. However, these databases and AI models follow different data formats and design standards, making it difficult to process information with the uniform codes. In this case, we develop data processing tools specific to different databases and AI models in the web service. Considering that huge calculation resources can possibly be required for AI models, it tends to be better to deploy AI models on a custom high-performance server.

e. LLM chat agent

LLM is integrated to provide a language query entry for basic chemistry knowledge and knowledge graph content. Developed knowledge graphs are preloaded into the LLM in the form of triples as chat context (Supplementary Triples S2). In this work, ChatGPT[10] is utilised as an example LLM. The OpenAI API is integrated into the knowledge graph framework with a Python library (https://github.com/openai/openai-python). The REST API documentation can be found on the OpenAI platform (https://platform.openai.com).

Supplementary Triples S2. Example triplets preloaded into the LLM.

<Arrhenius>	<Is-A>	<ReactionKineticsPhenomenon>
<Concentration Power Dependence>	<Is-A>	<ReactionKineticsPhenomenon>
<Eley-Rideal Surface Catalysis>	<Is-A>	<ReactionKineticsPhenomenon>
<Engulfment>	<Is-A>	<MolecularTransportPhenomenon>
<AnnularFlowReactor>	<relatesToFlowPattern>	<Gas-induced Annular Flow>
<Gas-induced Annular Flow>	<relatesToMolecularTransport>	<Engulfment>

<Concentration Power Dependence>	<hasFormula>	<mrow><br><mo>∏</mo><br><msup><br><mi>c</mi><br><mi>n</mi><br></msup><br></mrow>
<Engulfment>	<hasFormula>	<mfrac><br><mrow><br><msub><br><mi>c</mi><br><mtext>m</mtext><br></msub><br><mo>−</mo><br><mi>c</mi><br></mrow><br><msub><br><mi>t</mi><br><mtext>m</mtext><br></msub><br></mfrac>

6. Web deployment

Following the separation of concerns principle, knowledge graphs and developed agents are deployed separately, with minimal overlapping between functional units. The knowledge graph is dumped into a graph database, GraphDB, in this work with an exposed API for access. The SPARQL query is sent to this API for searching triples from the knowledge graph. All developed agents are deployed in the web service, Flask, with an API to invoke. These two sections are containerised with Docker to effectively manage, scale, and maintain the applications. A running GraphDB Docker container can support the accesses from multiple web service Docker containers. Supplementary Fig. S12 illustrates the web deployment pattern.

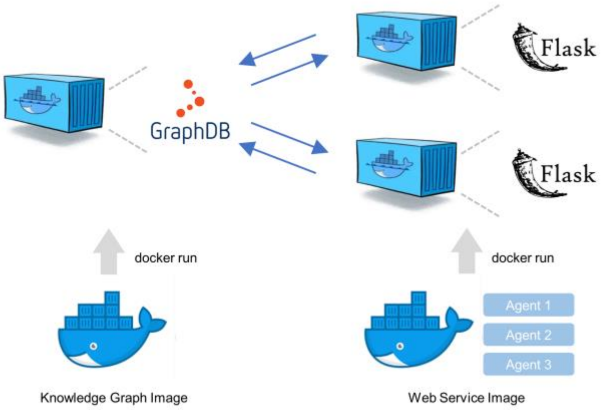

Supplementary Fig. S12 Deployment of knowledge graph and web service. Docker is implemented to build images for the knowledge graph and the web service with agents incorporated.

7. Experiment methods

In the TCR case, the ^1H NMR spectra of the BBAc product is shown as Supplementary Fig. S13. We established the HPLC calibration curve of BBA and BBAc as shown in Supplementary Figs. S14 and S15. For rapid analysis of the amidation reaction, we established the HPLC calibration curve of BAA as shown in Supplementary Fig. S16.

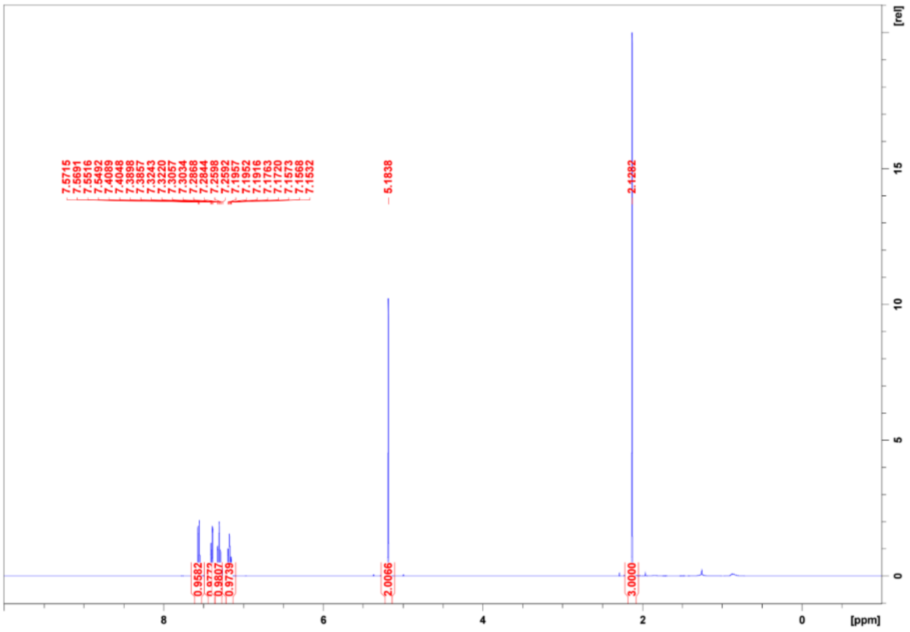

Supplementary Fig. S13 ^1H NMR (400 MHz, $CDCl_3$) spectra of the synthesised BBAc. ^1H NMR ($CDCl_3$): 7.57-7.55 (m, 1H), 7.41-7.39 (m, 1H), 7.32-7.28 (m, 1H), 7.20-7.15 (m, 1H), 5.18 (s, 2H), 2.13 (s, 3H). Multiplicities are reported as follows: s = singlet, d = doublet, m = multiplet, br = broad.

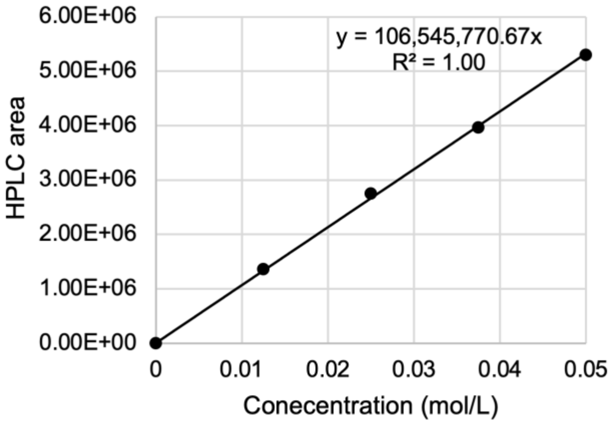

Supplementary Fig. S14 HPLC calibration curve of BBA.

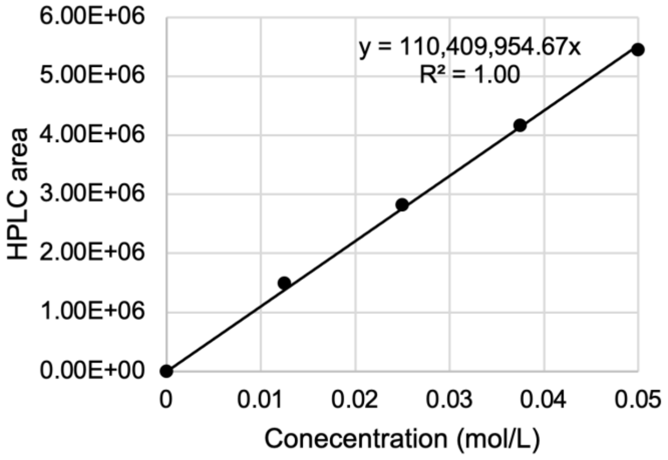

Supplementary Fig. S15 HPLC calibration curve of BBAc.

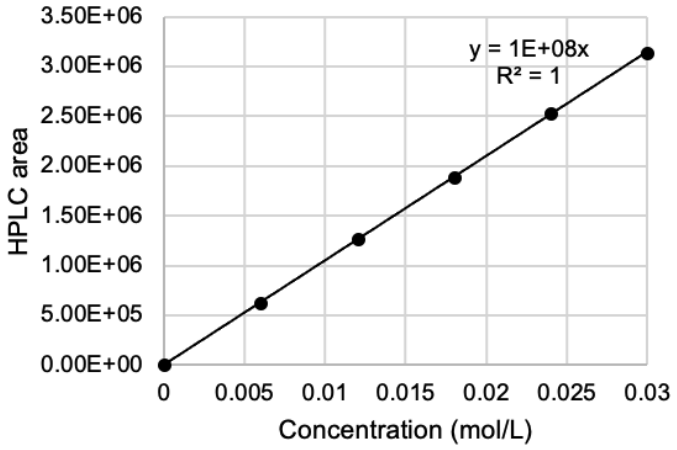

Supplementary Fig. S16 HPLC calibration curve of the analyte BAA.

References

1. Morbach, J., Wiesner, A. & Marquardt, W. OntoCAPE—A (re)usable ontology for computer-aided process engineering. *Comput. Chem. Eng.* **33**, 1546–1556 (2009).
2. Caprotti, O. & Carlisle, D. OpenMath and MathML: semantic markup for mathematics. *XRDS* **6**, 11–14 (1999).
3. Commenge, J.-M. & Falk, L. Villermaux–Dushman protocol for experimental characterization of micromixers. *Chem. Eng. Process. Process Intensif.* **50**, 979–990 (2011).
4. Kockmann, N., Kiefer, T., Engler, M. & Woias, P. Convective mixing and chemical reactions in microchannels with high flow rates. *Sens. Actuators B Chem.* **117**, 495–508 (2006).
5. Panić, S., Loebbecke, S., Tuercke, T., Antes, J. & Bošković, D. Experimental

approaches to a better understanding of mixing performance of microfluidic devices. *Chem. Eng. J.* **101**, 409–419 (2004).

6. Men, Y. *et al.* Determination of the Segregation Index to Sense the Mixing Quality of Pilot- and Production-Scale Microstructured Mixers. *Chem. Eng. Res. Des.* **85**, 605–611 (2007).
7. Frazier, P. I. A Tutorial on Bayesian Optimization. Preprint at https://doi.org/10.48550/arXiv.1807.02811 (2018).
8. Loh, W.-L. On Latin hypercube sampling. *Ann. Stat.* **24**, 2058–2080 (1996).
9. Pérez, J., Arenas, M. & Gutierrez, C. Semantics and complexity of SPARQL. *ACM Trans. Database Syst.* **34**, 16:1-16:45 (2009).
10. OpenAI *et al.* GPT-4 Technical Report. Preprint at https://doi.org/10.48550/arXiv.2303.08774 (2024).
